# Revealing innovative JAK1 and JAK3 inhibitors: a comprehensive study utilizing QSAR, 3D-Pharmacophore screening, molecular docking, molecular dynamics, and MM/GBSA analyses

**DOI:** 10.3389/fmolb.2024.1348277

**Published:** 2024-03-07

**Authors:** Abdelmoujoud Faris, Ivana Cacciatore, Radwan Alnajjar, Hadni Hanine, Adnane Aouidate, Ramzi A. Mothana, Abdullah R. Alanzi, Menana Elhallaoui

**Affiliations:** ^1^ LIMAS, Department of Chemical Sciences, Faculty of Sciences Dhar El Mahraz, Sidi Mohamed Ben Abdellah University, Fez, Morocco; ^2^ Department of Pharmacy, University ‘G. d’Annunzio’ of Chieti-Pescara, Chieti, Italy; ^3^ CADD Unit, PharmD, Faculty of Pharmacy, Libyan International Medical University, Benghazi, Libya; ^4^ School of Applied Sciences of Ait Melloul, Ibn Zohr University, Fez, Morocco; ^5^ Department of Pharmacognosy, College of Pharmacy, King Saud University, Riyadh, Saudi Arabia

**Keywords:** Janus kinases, FDA-drug, cytochrome P450, rheumatoid arthritis, immune system, interleukin, cancer

## Abstract

The heterocycle compounds, with their diverse functionalities, are particularly effective in inhibiting Janus kinases (JAKs). Therefore, it is crucial to identify the correlation between their complex structures and biological activities for the development of new drugs for the treatment of rheumatoid arthritis (RA) and cancer. In this study, a diverse set of 28 heterocyclic compounds selective for JAK1 and JAK3 was employed to construct quantitative structure-activity relationship (QSAR) models using multiple linear regression (MLR). Artificial neural network (ANN) models were employed in the development of QSAR models. The robustness and stability of the models were assessed through internal and external methodologies, including the domain of applicability (DoA). The molecular descriptors incorporated into the model exhibited a satisfactory correlation with the receptor-ligand complex structures of JAKs observed in X-ray crystallography, making the model interpretable and predictive. Furthermore, pharmacophore models ADRRR and ADHRR were designed for each JAK1 and JAK3, proving effective in discriminating between active compounds and decoys. Both models demonstrated good performance in identifying new compounds, with an ROC of 0.83 for the ADRRR model and an ROC of 0.75 for the ADHRR model. Using a pharmacophore model, the most promising compounds were selected based on their strong affinity compared to the most active compounds in the studied series each JAK1 and JAK3. Notably, the pharmacokinetic, physicochemical properties, and biological activities of the selected compounds (As compounds ZINC79189223 and ZINC66252348) were found to be consistent with their therapeutic effects in RA, owing to their non-toxic, cholinergic nature, absence of P-glycoprotein, high gastrointestinal absorption, and ability to penetrate the blood-brain barrier. Furthermore, ADMET properties were assessed, and molecular dynamics and MM/GBSA analysis revealed stability in these molecules.

## Introduction

Autoimmune diseases are characterized by an inappropriate immune system response against the body’s healthy cells and tissues (Lai and Dong, 2016; Hosseini et al., 2020). They encompass a heterogeneous group of conditions such as rheumatoid arthritis (RA), psoriasis, or type 1 diabetes (Elekofehinti et al., 2018; Tshiyoyo et al., 2022). These diseases result from a disruption of immune tolerance, leading to an abnormal self-reactive response. The JAK-STAT signaling pathway plays a key role in the activation and differentiation of immune cells. It is involved in transmitting external signals to cells through pro-inflammatory cytokines (Agrawal, n. d.; Pawluk et al., 2020). Janus kinases (JAK) are tyrosine kinases associated with cytokine receptors that, when activated, phosphorylate STAT transcription factors (Faris et al., 2023c).

Over the past 2 decades, significant strides have been achieved in the realm of pioneering therapies for autoimmune disorders, particularly within the field of biology. Notably, the strategic targeting of diverse cytokine pathways has demonstrated remarkable effectiveness in addressing a variety of medical conditions. For instance, the endorsement of anti-IL12/IL23 agents or more selectively tailored anti-IL23 treatments for afflictions such as psoriasis, psoriatic arthritis, and Crohn’s disease in both European and American regions has substantiated their efficacy and advantageous safety profiles (Lai and Dong, 2016). Nonetheless, despite those therapeutic breakthroughs, the inherent immunogenicity of therapeutic antibodies poses a persistent challenge, further exacerbated by the restriction that their administration is confined to parenteral routes. Promising avenues for the treatment of various immune disorders, including psoriasis, rheumatoid arthritis (RA), and inflammatory bowel disease (IBD), lie in the domain of JAK inhibitors, which are either already available on the market or are presently undergoing developmental stages (Gadina et al., 2018).

**FIGURE 1:**
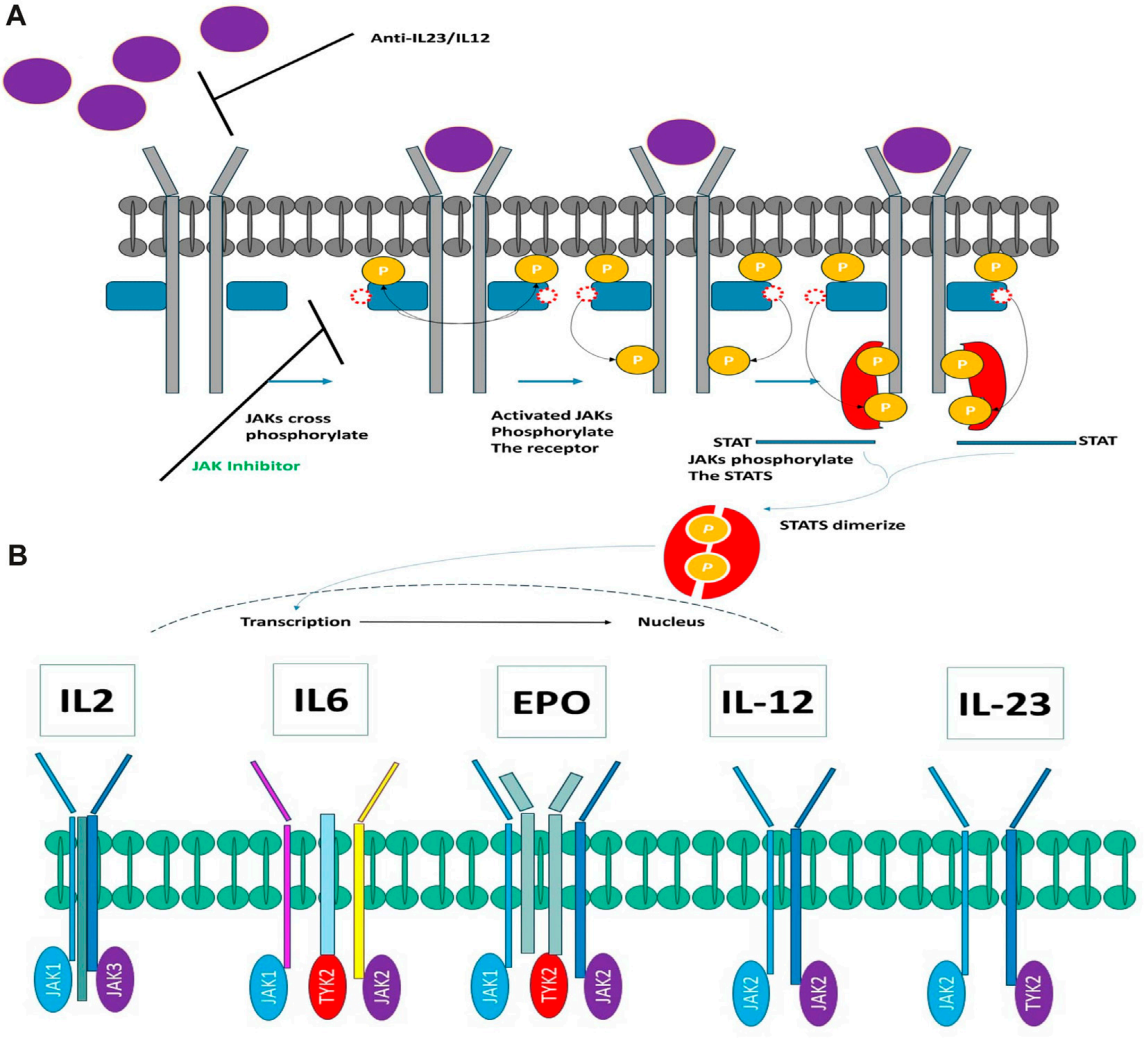
**(A)** Exploring the Intricacies of Cytokine Signaling: Unraveling Cellular Communication Pathways. **(B)** Heterodimers connect JAK kinases with cytokine receptors.

The JAK family encompasses four distinct members: JAK1, JAK2, JAK3, and TYK2, all of which belong to the category of tyrosine kinases. These kinases form associations with the intracellular domains of diverse cytokine and growth factor receptor chains. Activation of JAKs is instigated through ligand-induced conformational alterations within receptor complexes, setting off a phosphorylation cascade that ultimately activates members of the signal transducer and activator of the transcription (STAT) family (Kurdi and Booz, 2009). Once phosphorylated, STATs migrate into the cell nucleus, where they modulate gene expression in a manner contingent upon the specific ligand (see Figure 1A). Figure 1A elucidates the intricate process of cytokine signaling. Furthermore, it has been scientifically established that disparate cytokine receptors create specific heterodimers or homodimers in association with JAK enzymes (as depicted in Figure 1B). Each JAK, in turn, is affiliated with multiple receptors. Extensive research has elucidated that JAK1/JAK3 is reliant on γ-common chain cytokines, the JAK1/JAK2 complex is associated with interferon-gamma (INFγ), interleukin-6 (IL-6), and other gp130 cytokines. Meanwhile, the JAK1/TYK2 heterodimer binds to type I interferons and the IL-10 family of cytokines. Notably, JAK2 stands out by forming a homodimer on erythropoietin (EPO) and leptin receptors.

Figure 1B elucidates the intricate interconnection between JAK kinases and cytokine receptors in the form of heterodimers. In recent times, a multitude of JAK inhibitors have either garnered regulatory approval or are presently during clinical development (Figure 1B). These inhibitors manifest a spectrum of distinctive attributes. Ruxolitinib, representing the inaugural JAK inhibitor to make its foray into the market, operates as a JAK1/JAK2 inhibitor. It is harnessed for the therapeutic management of polycythemia vera and myelofibrosis (McKeage, 2015). Meanwhile, Tofacitinib, the pioneer JAK inhibitor adopted for the management of autoimmune maladies, functions as a pan-JAK inhibitor, with a degree of selectivity directed toward JAK1, JAK2, and JAK3 (Emery et al., 2018; Faris et al., 2024).

**FIGURE 2:**
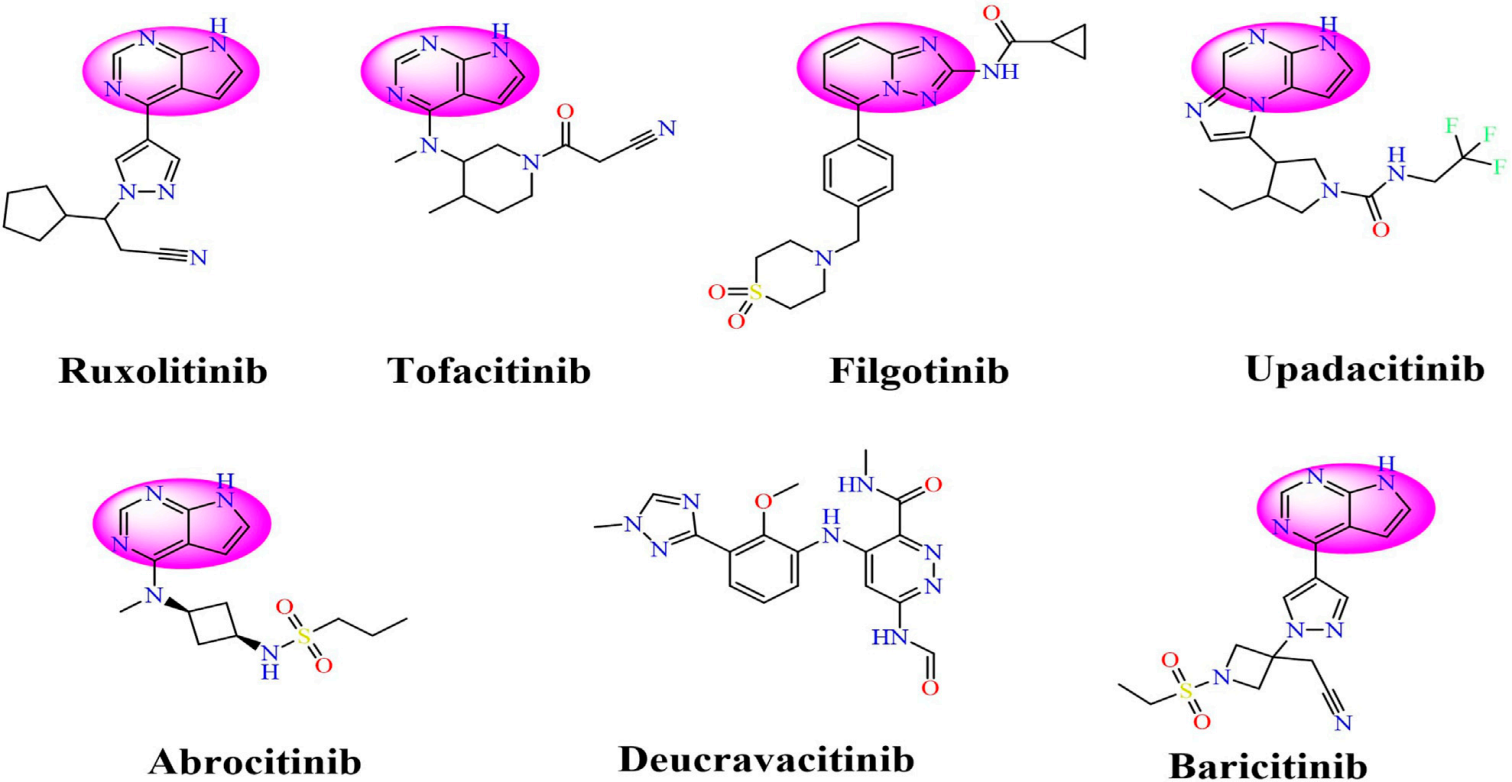
JAKs inhibitors.

Drawing from the accumulated wealth of knowledge concerning JAKs and their intricate interactions with cytokine receptors, pharmaceutical enterprises have been diligently engaged in the development of compounds featuring refined selectivity profiles (Figure 2). These novel compounds are designed to efficaciously regulate the disparate signaling pathways governed by various cytokines. A notable exemplar of such endeavors includes the development of JAK1 inhibitors, typified by Filgotinib, Baricitinib, Upadacitinib, and Abrocitinib, along with TYK2 inhibitors like BMS-986165. These compounds are presently undergoing clinical evaluation and hold promise as potential therapeutic agents for a spectrum of autoimmune maladies, encompassing rheumatoid arthritis (RA), psoriasis, and Crohn’s disease (6,7).

JAK3 and JAK1 are involved in the signaling of numerous pro-inflammatory cytokines. Their inhibition serves to thwart their pro-inflammatory activity. These two kinases play a role in the activation and proliferation of T and B lymphocytes (Henderson Berg et al., 2022). Their blockade reduces the activation of the immune system in autoimmune diseases. Inhibiting JAK3 or JAK1 decreases the production of pro-inflammatory cytokines such as interleukin-6 and interleukin IL-17, two key cytokines in rheumatoid arthritis (Henderson Berg et al., 2022; Kotyla et al., 2022). JAK3 inhibitors have demonstrated significant efficacy in treating rheumatoid arthritis by reducing inflammation and symptoms (Boyadzhieva et al., 2022; Chen et al., 2023; Sardana et al., 2023). Their immunomodulatory mechanism of action makes them a therapeutic option for cases resistant to conventional treatments like DMARDs and anti-TNF agents. Gamma is a common subunit of many cytokines such as interleukin IL-2, IL-4, IL-7, IL-9, IL-15, and IL-21 (Prasad et al., 2023). When these cytokines bind to their receptor, they activate the JAK/STAT signaling pathway through the phosphorylation of gamma by receptor-associated JAK kinases (Choy, 2019).

In the case of interleukin IL-2, IL-4, IL-9, and IL-21, it is primarily JAK1 that phosphorylates gamma (Menet et al., 2013). For interleukin IL-7 and IL-15, it is JAK3 that is responsible for gamma phosphorylation (Menet et al., 2013). This phosphorylation creates binding sites for STAT proteins, which, once activated, stimulate the transcription of genes involved in the proliferation and differentiation of lymphocytes (Menet, 2018; Morris et al., 2018). Inhibition of JAK1 or JAK3 by medications thus blocks the phosphorylation of gamma induced by associated cytokines. This prevents the activation of downstream STAT pathways and, therefore, the pro-inflammatory immune response mediated particularly by T and B lymphocytes. JAK1 and JAK3, through gamma phosphorylation, play a central role in the signal transduction of numerous pro-inflammatory cytokines, explaining their involvement in autoimmune diseases (Menet, 2018). Inhibition of these two kinases allows for the modulation of immune system activation in autoimmune diseases through similar effects on the JAK/STAT pathway. By blocking these proteins, inflammation can be reduced, and disease progression slowed, providing an alternative for patients for whom standard treatments are ineffective.

The JAKs uniquely possess an integrated pseudokinase domain (JH2) that regulates the adjacent kinase domain (JH1). The therapeutic targeting of JH2 domains has been less thoroughly explored and may present an avenue to modulate the JAKs without the adverse effects associated with targeting the adjacent JH1 domain (Henry and Jorgensen, 2023; Rodriguez Moncivais et al., 2023). The potential of this strategy was recently demonstrated with the FDA approval of the TYK2 JH2 ligand deucravacitinib for treating plaque psoriasis. In this light, the structure and targetability of the JAK pseudokinases are discussed, in conjunction with the state of development of ligands that bind to these domains (Grant et al., 2023; Henry and Jorgensen, 2023).

The obvious technique for developing small-molecule JAK3 inhibitors is to target the JAK3 kinase domain’s catalytic ATP-binding site (JH1). There have been numerous ATP-competitive JAK3 inhibitors produced (Christy, 2020). Signals communicated by the JAK3 protein affect the growth and maturation of types of white blood cells known as T lymphocytes, B lymphocytes, and natural killer cells, which are responsible for immune system regulation. As a result, among the JAK kinases, JAK1 is the primary activator of STAT3 phosphorylation and signaling (Tan et al., 2015). The manner of inhibitor binding in JAK1 was remarkably similar to that observed in JAK2, emphasizing the difficulties in designing JAK-specific inhibitors that target the ATP-binding site (Williams et al., 2009; Virtanen et al., 2023). Nonetheless, variations in the ATP-binding sites of JAK1 and JAK2 were observed, providing a foundation for the rational design of JAK2- and JAK1-specific inhibitors (Le et al., 2023).

Tofacitinib was initially created as a JAK3 inhibitor to prevent organ rejection, but additional research discovered that it is also a powerful inhibitor of JAK1 and JAK2 (Tan et al., 2015). Tofacitinib is a medication used to treat certain autoimmune diseases, notably rheumatoid arthritis (X. Yang et al., 2021). It belongs works by modulating the immune system to reduce inflammation associated with these conditions (Hosseini et al., 2020). Tofacitinib has been approved by various regulatory agencies, including the Food and Drug Administration (FDA) in the United States, for use in the treatment of rheumatoid arthritis and other autoimmune disorders (Mogul et al., 2019; Roskoski Jr, 2023).

To expedite the drug synthesis process and achieve the desired target pathways, Computer-Aided Drug Design (CADD) approaches are employed, utilizing potent and diverse methodologies (Nascimento et al., 2022; Zhu et al., 2023).

In many instances, the preference for experimental techniques as the superior option in molecular dynamics can be observed (Kukol, 2014). For instance, spectroscopy is employed to investigate bond vibrations, and electrophysiology is utilized to scrutinize the opening and closing of ion channels. Nonetheless, substantial progress has been achieved in theoretical methods over the past few decades, resulting in numerous domains where modeling and simulation either offer a higher level of detail or are more efficient than initiating a new experimental undertaking. Molecular docking, endeavor revolves around the prediction of the structure (or structures) of the intermolecular complex that arises from the interaction of two or more molecules (Leach, 2001). The utilization of docking is widespread for proposing the binding modes of protein inhibitors. Most docking algorithms can generate a substantial number of potential structures, necessitating a method for scoring each structure to identify those of utmost significance. Consequently, the ‘docking problem’ is primarily concerned with the generation and assessment of plausible structures for intermolecular complexes.

Concerning this investigation, the 2D-QSAR, 3D pharmacophore model was utilized and rigorously validated through robust statistical methods, demonstrating its significant efficacy in obtaining more potent inhibitors against JAK1 and JAK3 (Kapetanovic, 2008; Nascimento et al., 2022; Faris et al., 2023c). This was achieved through screening an extensive database, which included a collection of predicted compounds and natural substances obtained from PubChem and ZINCData. Among the compounds identified in this study, Baricitinib and Ruxolitinib stood out as the JAK inhibitors, underscoring the importance of the pharmacophore model in obtaining JAK inhibitors. However, in subsequent investigations, new inhibitors were subject to a stringent selection process involving docking studies and ADMET evaluation, resulting in the removal of certain compounds from consideration. This step was crucial in ensuring favorable pharmaceutical properties. Molecular dynamics and MM/GBSA analyses were employed to confirm the stability of these molecules, as they exhibited potent affinities, indicating robust binding. In this comparative study, Tofacitinib was used as a reference, given its established efficacy in the treatment of rheumatoid arthritis. In the identification of molecules, a notable observation is the prevalence of compounds featuring a pyrazole-pyrimidine chain. This observation aligns with previous studies, reinforcing the pivotal role played by such molecules in the inhibition of JAKs. The consistent recurrence of this structural motif underscores its significance in influencing the binding affinity and inhibitory potential of compounds targeting JAK proteins. This finding adds valuable insights to the growing body of knowledge supporting the design and development of effective JAK inhibitors. The newly discovered inhibitors may represent promising candidates for *in vitro* synthesis as inhibitors against JAK1 and JAK3.

## Methods and materials

### Dataset

In this study, an ensemble of data was utilized based on previous work involving Cyanamide-Based Janus Kinase 1 and 3 (JAK1, JAK3) compounds (Casimiro-Garcia et al., 2018). The objective was to evaluate the inhibitory activity of these compounds against the target kinases. The dataset comprised 28 molecules, and the inhibitory activity of each molecule was experimentally measured in terms of IC_50_, expressed in nanomolar (nM) units. To facilitate result comparison and analysis, the IC_50_ values were converted to pIC_50_ using the formula -log (IC50 * 10^–9^). The obtained pIC_50_ values for each molecule were recorded (See the Supplementary Table S1). The pIC_50_ essentially represents the inhibitory activity of each compound, with higher values indicating greater inhibitory activity. These were further utilized to develop pharmacophore models. For the implementation of the 2D-QSAR model, the molecules were divided into 23 compounds for the training set and 5 for the test set.

### Calculation and selection of molecular descriptors

Initially, the SMILES representations of the examined compounds underwent conversion into Structure Data File (SDF) format. Subsequently, the SDF files were submitted to the PaDel software for (Yap, 2011) the computation of 1D, 2D, and 3D molecular descriptors, employing the MM2 force field for minimization. A comprehensive set of 1135 molecular descriptors were then quantified.

### Model development

In this investigation, four distinct modeling approaches—namely, Multiple Linear Regression (MLR), and ANN were employed to scrutinize the relationship between the molecular structure and the activity of the compounds under examination. For these models, criteria indicative of predictive and statistically acceptable models included a substantial F -value ( F>0.33 ), a determination coefficient $\left(R^{2}\right)$ , a mean squared error (MSE), an adjusted coefficient $\left(R_{adj}^{2}\right)$ , a lower BIC (Bayes information criterion), a higher $R^{2}$ , an increased post-probability, and a significance level (*p*-value) less than or equal to 5% (Faris et al., 2023a)

$$R_{cu}^{2}=1-\frac{\sum_{i-1}^{n}\;{\left(Y_{abs}-Y_{cal}\right)}^{2}}{\sum_{i=1}^{n}\;{\left(Y_{pas}-{\bar{Y}}_{cal}\right)}^{2}},$$ (1)

$$R_{adj}^{2}=\frac{\left(n-1\right)\times R^{2}-p}{n-1-p},$$ (2)

$$MSE=\frac{1}{n}\sum_{i=1}^{n}\;{\left(Y_{obs}-Y_{cal}\right)}^{2},$$ (3)

$$F=\frac{\sum {\left(Y_{\text{cal}\;}-{\bar{Y}}_{\text{mean}\;}\right)}^{2}}{\sum {\left(Y_{\text{abs}\;}-{\bar{Y}}_{\text{cal}\;}\right)}^{2}}\times \frac{n-p-1}{p},$$ (4)

$Y_{\text{obs}\;}$ represent the observed values, ${\bar{Y}}_{cal}$ signifies the predicted response values, and ${\bar{Y}}_{\text{cal}}$ denotes the average value derived from either observed or predicted data. The variable p refers to the number of explanatory variables, while n stands for the total number of compounds in the dataset.

## MLR

Due to its reliability and simplicity, the MLR approach is commonly utilized in Quantitative Structure-Activity Relationship (QSAR) research for the identification of molecular descriptors (Roy et al., 2016). The foundational premise of MLR lies in the notion that the biological activity of JAKs inhibition (expressed as pIC_50_) is linearly associated with a specific set of molecular descriptors, as depicted by the equation in Eq. 6.

$$Y=a_{0}+\sum_{i=1}^{n}\;\left(a_{i}X_{i}\right),$$ (5)

where n signifies the number of molecular descriptors, $X_{i}$ represents individual molecular descriptors, Y denotes the predicted biological activity, $a_{0}$ stands for the constant term in Eq. 6, and $a_{i}$ represents the coefficients associated with the respective molecular descriptors.

## ANN

**FIGURE 3:**
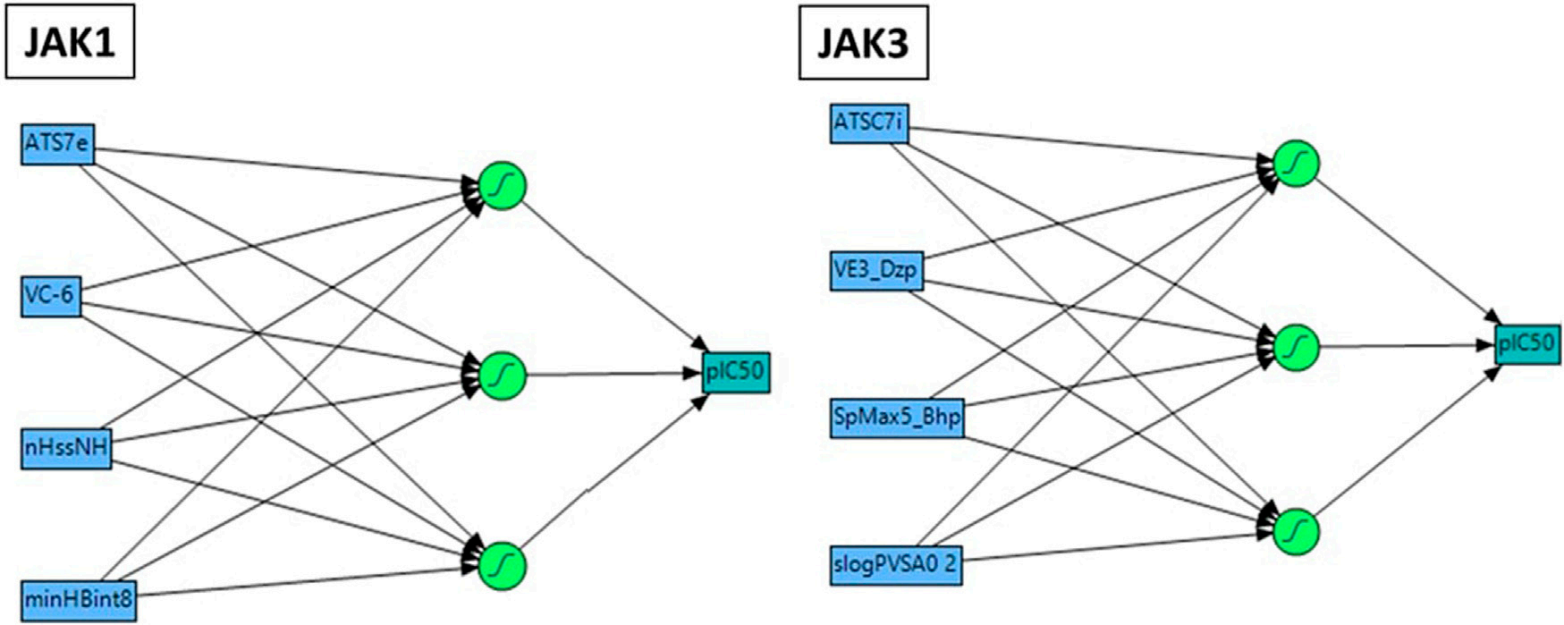
Neural network Architecture for molecular activity prediction with a 4-2 Configuration: Descriptor input, 2 neuron hidden layer, and activity output.

The nonlinear modeling approach known as ANN, comprising an input layer, one or more hidden layers, and an output layer, has found extensive application in QSAR research (Prado-Prado et al., 2010). In this study, we constructed a QSAR model utilizing the ANN technique to validate the precision of the pIC_50_ values obtained from preceding models. The employed configuration involved the use of sigmoid as the hidden layer transfer function, and training was executed through the scaled conjugate gradient algorithm employing a feed-forward method (Figure 3). The pIC_50_ experiment served as the output layer, encompassing the predicted pIC_50_ activity levels. The input layer consisted of a neuron set equal to or less than the number of molecular descriptors selected by the Multiple Linear Regression model. Striking a balance in the number of hidden layers is crucial, as an excess leads to overfitting, while too few compromises fault tolerance and generalizability. Consequently, the outlined strategy led to the implementation of a 4–3–1 network design in the present investigation.

### Internal validation

The QSAR models generated by MLR and ANN were internally validated using leave-one-out cross-validation (LOOCV) as the chosen method. The $R^{2}\text{cv}$ coefficient value was computed using Eq. 10. A criterion for model predictiveness was established, where an $R_{cu}^{2}$ exceeding 0.5 was considered indicative of a reliable predictive model. This threshold suggests that the internal predictions made by the model are accurate (Golbraikh and Tropsha, 2002).

$$R_{cv}^{2}=1-\frac{\sum {\left(Y_{\text{cde}\;}\left(\text{train}\right)-Y_{\text{cal}\;}\left(\text{train}\right)\right)}^{2}}{\sum {\left(Y_{\text{cis}\;}\left(\text{train}\right)-{\bar{Y}}_{\text{col}\;}\left(\text{train}\right)\right)}^{2}}.$$ (6)

where $Y_{\text{obs}\;}$ (train) observed value, ${\text{Y}}_{\text{cal}}($ train) the Loo-cv response prediction value, and ${\bar{\text{Y}}}_{\text{cal}}($ train) denotes the average value of the observed or predicted data.

### External validation

The QSAR models developed for predicting the activities of the compounds in the test set were utilized for external validation. The assessment of external validation involved calculating the coefficient $R_{\text{test}\;}^{2}$ , which represents the correlation between the actual pIC_50_ values and the predicted pIC_50_ values after incorporating the test set. The external predictive capability of the QSAR models in forecasting the activity of the test set compounds was gauged through this coefficient. To evaluate the efficacy of $R_{\text{test}\;}^{2}$ in external validation, the criteria outlined by Golbraikh and Tropsha were applied (Golbraikh and Tropsha, 2002). Hence the if $R_{\text{test}\;}^{2}$ exceeds 0.5, the model is considered statistically robust for prediction and is deemed suitable for application with new external data (Hadni and Elhallaoui, 2020; Aouidate et al., 2021) (Table 1).

**TABLE 1:** Model evaluation criteria.

Parameter	Formula	Threshold value
**Q** ^**2**^	$Q^{2}=1-\frac{\sum {\left(Ypred\left(test\right)-Y\left(test\right)\right)}^{2}}{\sum {\left(Y\left(test\right)-{\underline{Y}}_{tr}\right)}^{2}}$	>0.5
**R** ^**2**^	The coefficient of determination for the graph of expected *versus* seen for the test set	>0.6
$\left|R_{0}^{2}-{R^{\prime }}_{0}^{2}\right|$		<0.3
$r_{0}^{2}$	r^2^ at zero intercept	
${R^{\prime }}_{0}^{2}$	r_'0_^2^ for the plot of observed against projected activity for the test set at zero intercept	
$\frac{R^{2}-R_{0}^{2}}{R^{2}}$		<0.1
$\frac{R^{2}-R_{0}^{\prime 2}}{R^{2}}$		<0.1
**k**	The slope of the plot of observed v/s projected activity at the intercept	0.85 < k < 1.15
**k'**		0.85 < k' < 1.15

### Y-randomization test

In this analysis, the Y-randomization test was employed to eliminate the possibility of a random correlation between molecular descriptors and the biological activities of the compounds under investigation. The data was divided into a training set and a test set, with the training set utilized for conducting the Y-randomization test (Rücker et al., 2007). The Y-randomization test establishes the validity of the QSAR model by comparing the average random correlation coefficient $\left(R_{r}^{2}\right)$ derived from randomized models to the correlation coefficient of the original non-random model, which includes the Multiple Linear Regression (MLR), and Artificial Neural Network (ANN). If $R_{r}^{2}$ of randomization model is less than $R_{r}^{2}$ and $cR_{p}^{2}$ exceeds 0.5, the Y-randomization test indicates that the QSAR model is valid and not a result of random chance (Roy and Mitra, 2011).

$$cR_{p}^{2}=R^{*}\sqrt{R^{2}-{\left(\;\text{average}\;R_{\text{rand}\;}\right)}^{2}},$$ (7)

### DoA

Defining the DoA of QSAR models is imperative as compounds falling outside this domain may not be considered reliable for prediction. In this study, the reliability of predicting the activities of the examined compounds by DoA models, utilizing leverage values $\left(h^{i}=x_{i}^{T}{\left(X^{T}X\right)}^{-1}x_{i}\right)$ , with $\left(i=1,2,\ldots ,n\right)$ , for each compound, xi represents a vector describing the compound, X is the matrix of K descriptor values of the model for n × (K-1) compounds in the training set, and the exponent. T denotes matrix/vector transposition (Gramatica, 2007), was assessed. The Williams plot, utilizing pIC_50_ and descriptors in the training and test sets specifically chosen for the MLR model, was employed to calculate the applicability domain within a rectangular area and the degree of leverage $h^{*}\left(h^{*}=3\times \frac{\left(k+1\right)}{n}\right)$ , with k specified descriptors in the model and n specified compounds in the training set (Netzeva et al., 2005). A compound is considered outside the application range if its leverage effect (h) surpasses its alert leverage (j), indicating a negative impact on the established model.

### Drug likeness and ADMET prediction

In this study, drug-likeness was employed to identify compounds suitable for medicinal use, adhering to three critical principles: Lipinski’s, Veber’s, and Igan’s. Additionally, properties related to the central nervous system (CNS), such as the number of rotatable bonds (n-ROTB), blood-brain barrier (BBB) permeability, P-glycoprotein substrate status, and topological polar surface area (TPSA), were considered for assessing the characteristics of the studied compounds. The SwissADME web tool (Swiss Institute of Bioinformatics, Switzerland) was utilized for the analysis of drug-like properties (Azzam, 2023). Subsequently, the AdmetSAR 1.0 online program (Cheng et al., 2012), was employed to evaluate the Absorption, Distribution, Metabolism, Excretion, and Toxicity (ADMET) properties of the investigated compounds (Cheng et al., 2012). Parameters such as human intestinal absorption (HIA), Caco-2 permeability, aqueous solubility (LogS), and subcellular localization were assessed for absorption and distribution. The metabolism aspect involved an examination of common cytochrome P450 isoforms (e.g., CYP1A1, CYP2D6, CYP2C19, CYP3A4, etc.). Toxicological factors, including *Salmonella*/microsome (AMES) toxicity, biodegradation, carcinogenicity, fish toxicity (pLC50, mg/L), *Tetrahymena* pyriformis toxicity (pIGC50, ug/L), acute oral toxicity, and rat acute toxicity (LD50, mol/kg), were also considered.

### Biological activities (BA)

For a more in-depth exploration of the biological activities of the studied compounds, PASS online employed the Way2Drug server web tool. Utilizing PASS online, the biological roles, mechanisms of activity, and potential therapeutic effects of the compounds were elucidated. The platform predicts over 4,000 different types of biological activity by examining structure-activity relationships within approximately 250,000 physiologically active compounds, spanning prescription medicines, therapeutic candidates, hazardous chemicals, and drug leads (Lagunin et al., 2000). Upon submitting the selected compounds to PASS online, the platform assessed the likelihood of various therapeutic effects, such as cholinergic, antioxidant, anti-inflammatory, etc., using probable activity (Pa, probability to be active). Pa values, denoting the percentage likelihood of activity, ranged from 0.0001 to 1.000. This facilitated the identification and quantification of potential therapeutic effects associated with the studied compounds.

### Pharmacophore hypothesis

The pharmacophore hypothesis, a well-established framework in pharmaceutical chemistry, is pivotal for the identification and modeling of critical interactions between a drug molecule and its biological target. This concept is underpinned by the understanding that specific structural or chemical features of a molecule hold significant sway over its biological activity (Yang et al., 2010; Faris et al., 2023c). The advanced Schrödinger software, in its 2021 iteration, equips researchers with sophisticated tools for crafting and validating pharmacophore hypotheses. These tools harness insights into molecular interactions, encompassing hydrogen bonds, electrostatic interactions, and hydrophobic interactions (Mali et al., 2022).

**FIGURE 4:**
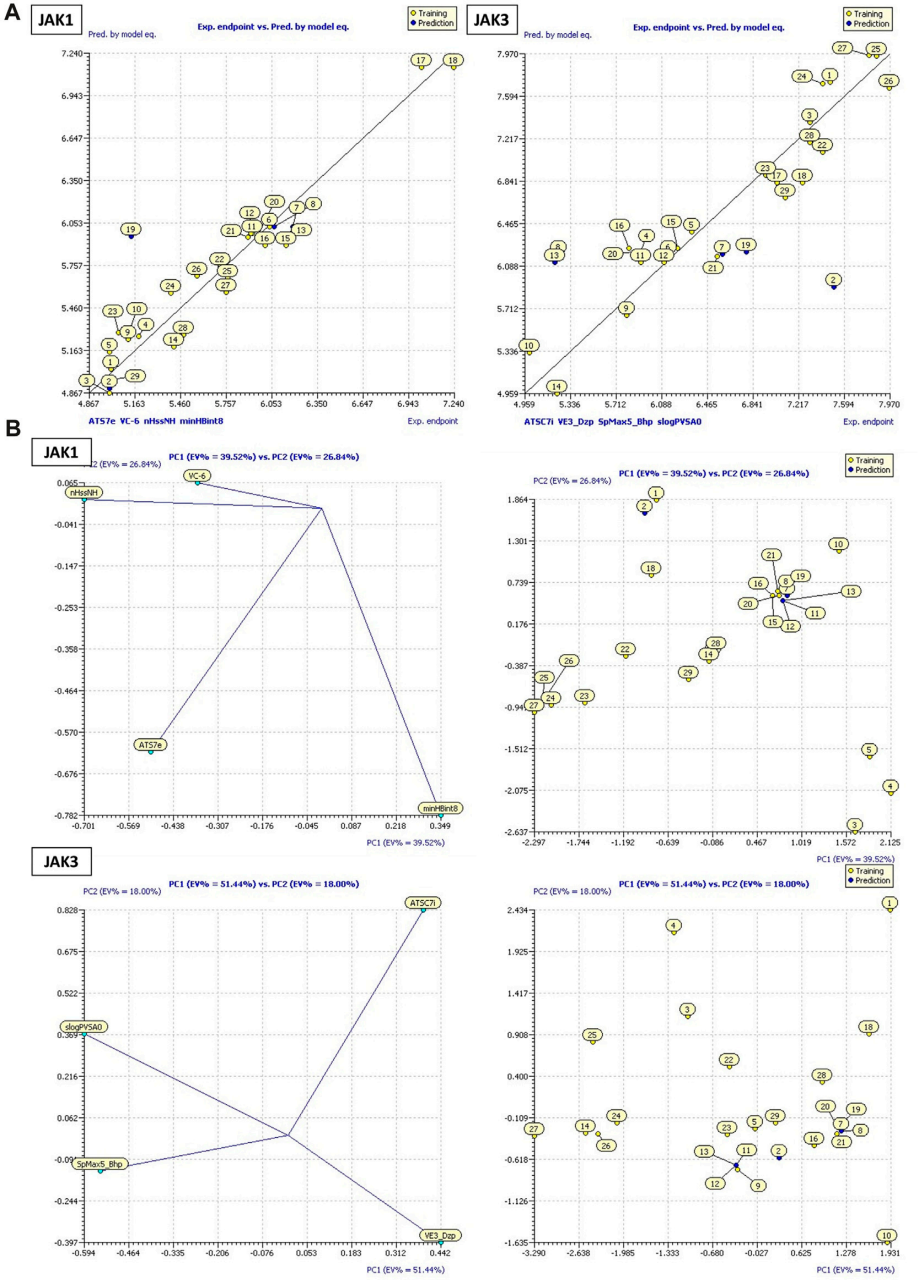
**(A)** Plots of MLR models. **(B)** Principal Component Analysis (PCA) Plots for Descriptors and Molecules in a Series Study.

**FIGURE 5:**
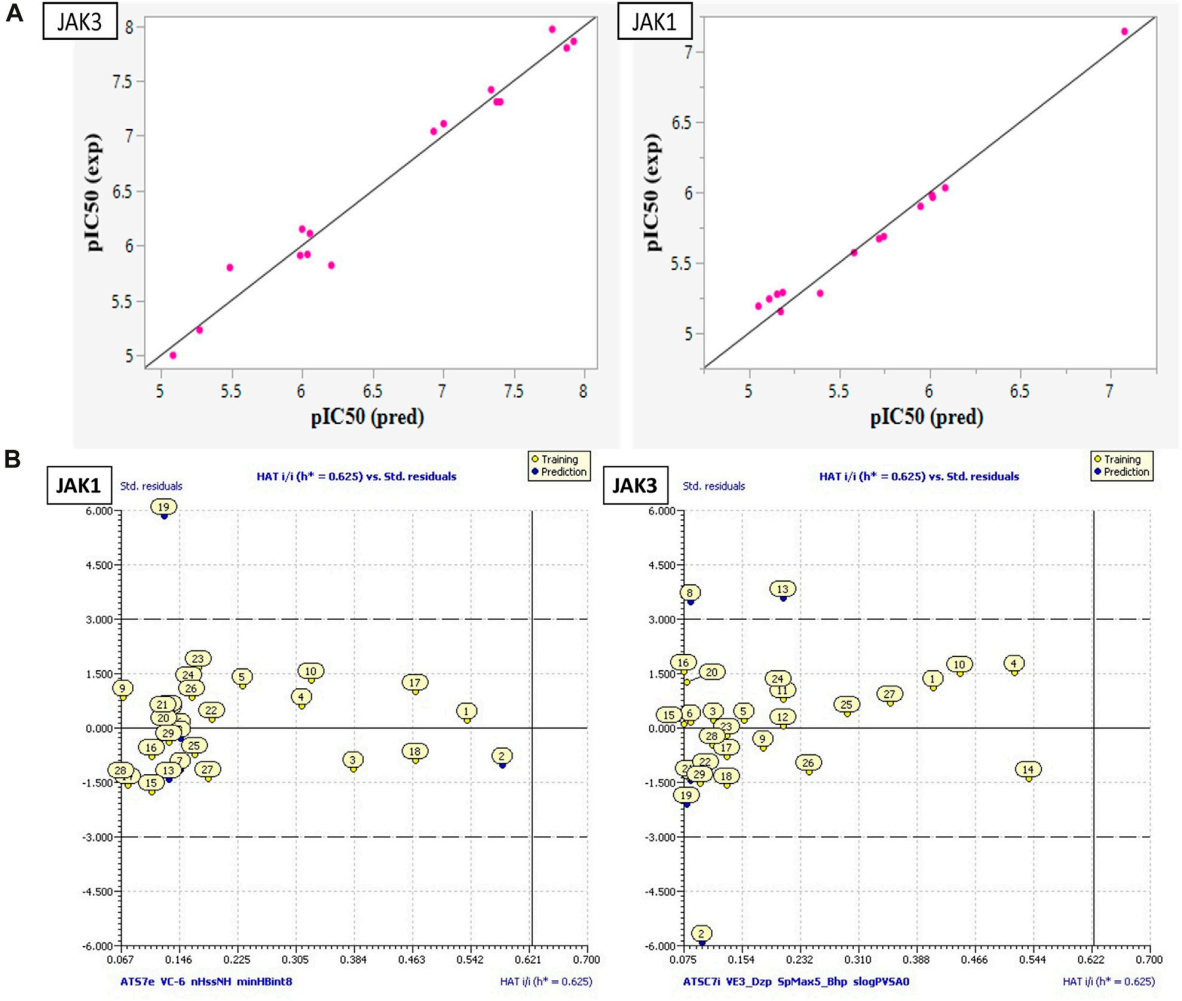
**(A)** Plot ANN models developed for both JAK1 and JAK3. **(B)** The William plot of AD.

In the context of pharmacophore modeling, ligand chemistry underwent meticulous normalization and extrapolation through the automated PHASE process. This approach aligns ligands based on their optimal arrangement and shared properties, as exemplified in Figures 4, 5. Subsequently, these rigorously prepared ligands were incorporated into the Maestro workspace (*Schrödinger Release 2021–1; Maestro, Schrödinger, LLC: New York, NY, USA,* 2021). Their experimental binding affinities, expressed as pIC50 values, were instrumental in classifying compounds as either active or inactive. An active compound met the criteria of a pIC_50_ value exceeding 6.5, while inactivity was associated with a binding affinity exceeding 10 µM or a pIC_50_ value below 6.5. To ensure meaningful matches, the assumption requirement necessitated a minimum of 50% of active compounds to be met, with a preference for a minimum of five features for a successful match. Most assumption criteria retained their default settings, except for donor and negative molecules, where ionic features were assigned a value of 1 to guarantee compatibility between acceptor and negative features.

### Molecular docking

#### Preparation of compounds

The compounds employed in the pharmacophore model and molecular docking process are constructed as SMILES representations. The Ligpred function within Maestro is utilized to transform these representations from a two-dimensional (2D) format to a three-dimensional (3D), followed by preprocessing of the molecular structures. This procedure further encompasses the generation of multiple poses for each structure. This comprehensive function not only facilitates the conversion to 3D but also ensures the structural readiness for subsequent docking simulations (Alamri et al., 2021; Faris et al., 2023c).

#### Docking software

The computational data about the compounds were obtained through *in silico* techniques using the Maestro and AutoDock Vina software applications, both of which were accessed through the DIA-DB platform (http://bio-hpc.eu/software/dia-db/). Maestro, a software product developed by Schrödinger, utilizes the Glide scoring function, which systematically assesses a wide array of interactions within the ligand-protein complex while simultaneously mitigating any steric hindrances, ultimately yielding docking scores [26]. On the other hand, AutoDock Vina employs an empirical scoring function that primarily takes into consideration simple contact terms and the influences of lipophilic and metal-ligand interactions within the ligand-protein complex to estimate the Gibbs free binding energy between the ligand and the protein [5,6].

#### Preparation of enzymes

The crystallographic data for the enzymes, namely, JAK1 (PDB ID: 3EYG) and JAK3 (PDB ID: 3LXK), as well as for the JAK3-selective inhibitor Tofacitinib, were procured through the Maestro software platform. Furthermore, for the Tofacitinib inhibitor, the PDB ID 4Z16, previously employed in prior research, was utilized. In the subsequent preparation steps, the protein preparation wizard within Maestro was employed to render the enzymes amenable to molecular docking (Manual, n. d.; *Schrödinger Release 2021–1; Maestro, Schrödinger, LLC: New York, NY, USA,* 2021). This process entailed the removal of all cofactors and water molecules, followed by structural optimization. The wizard effectively addressed any structural anomalies, ensuring that the enzyme structures were optimally configured for subsequent structural-based virtual screening (Faris et al., 2023b).

#### Protein-ligand interaction

Two essential components are required in the docking process: the grid file and the ligand file. To generate the grid file, the receptor grid generation tool within Maestro was utilized, employing the default parameters. Subsequently, protein docking was conducted employing the glide HTVS method (Alamri et al., 2021), where the prepared ligands were docked against the previously generated grid files specific to JAK1 and JAK3. The docking score is a relative measure corresponding to the change in Gibbs free energy (ΔG) associated with the interaction between the protein and the compound. A more negative docking score signifies increased stability, indicating a stronger binding affinity of the compound to the protein. The assessment of ΔG is influenced by various interactions within the protein-compound complex, encompassing hydrophobic interactions and hydrogen bonds among others.

#### Molecular dynamics

Molecular dynamics (MD) simulations were conducted using the GROMACS MD engine. Input files for these simulations were meticulously constructed with the assistance of CHARMM-GUI {Citation}, employing the CHARMM36 force field to dictate the system’s behavior (Jo et al., 2008; Huang and MacKerell Jr, 2013). The system was encapsulated within a cubic box and hydrated utilizing the TIP3P water model, with an added 10 Å buffer zone. NaCl salt molecules were introduced at a concentration of 0.15 M to maintain electrostatic neutrality, their placement was guided by the Monte Carlo method (Underwood and Greenwell, 2018; Yagasaki et al., 2020). The system’s energy was meticulously minimized over 250,000 steps via a gradient descent method. Following the energy minimization, the system underwent a crucial equilibration phase, residing in a constant atom number, volume, and temperature (NVT) ensemble at a controlled temperature of 310 K for a substantial 50 nanoseconds. Subsequently, the system embarked on unrestricted MD simulations spanning 100 nanoseconds, adhering to a constant number of atoms, pressure, and temperature (NPT) ensemble, with temperature and pressure set at 310 K and 1 atm, respectively. Trajectory analysis of the MD simulations was carried out using the sophisticated Visual Molecular Dynamics (VMD) software, specifically its 2020 version. In the context of the molecular dynamics (MD) simulations involving Tofacitinib and its interaction with the JAK3 target, it is important to note that the details of this specific MD simulation were introduced in a previous research endeavor. This previous work provides a comprehensive account of the methodology and parameters used for the MD simulation involving Tofacitinib and JAK3, thus serving as a foundational reference for the current study (Faris et al., 2023c). This in-depth analysis was pivotal in scrutinizing the system’s stability and deriving crucial parameters, including but not limited to the root-mean-square deviation (RMSD), root-mean-square fluctuation (RMSF), radius of gyration (RoG), protein solvent-accessible surface area (SASA), hydrogen bond interactions, and Principal Component Analysis (PCA) (Barz et al., 2019).

#### Fel and PCA

Biomolecular processes, such as molecular folding or aggregation, can be elucidated by considering the molecule’s free energy. In this context, the Boltzmann constant (kB) plays a pivotal role, with the probability distribution of the molecular system along a specific coordinate, denoted as R, being represented by P. Pmax signifies the maximum probability within this distribution, and its subtraction ensures that δG (the change in Gibbs free energy) equals zero at the lowest free energy minimum. $\Delta G\left(R\right)=-k_{B}T\left[\ln\;P\left(R\right)-\ln\;P_{\max}\right]$ . A selection of commonly employed order parameters for R includes the root mean square deviation (RMSD), radius of gyration (Rgyr), count of hydrogen bonds, native contacts, and principal components. These order parameters serve as the basis for plotting the free energy, resulting in a reduced free energy surface (FES) when considering two of them simultaneously. Principal component analysis (PCA), alternatively referred to as covariance analysis or essential dynamics analysis, shares commonalities with clustering techniques, as it aids in the discernment of significant features within extensive molecular trajectories. PCA, however, specializes in the identification of the most pronounced motions occurring within the system.

#### RMSD and RMSF

The Root Mean Square Deviation (RMSD) for specific atoms within a molecule concerning a reference structure, denoted, is computed as follows (Nicosia and Stracquadanio, 2008):

$$\text{RMSD}\left(t\right)={\left[\frac{1}{M}\sum_{i=1}^{N}\;m_{i}{\left|r_{i}\left(t\right)-r_{i}^{\text{ref}\;}\right|}^{2}\right]}^{\frac{1}{2}},$$ (8)

where M = Σi mi and **r**i (t) is the position of atom i at time t after least square fitting the structure to the reference structure.

The Root Mean Square Fluctuation (RMSF) serves as a metric for quantifying the disparity between the position of a particle, denoted as i, and a designated reference position (Islam et al., 2021):

$${\text{RMSF}}_{i}={\left[\frac{1}{T}\sum_{t_{j}=1}^{T}\;{\left|r_{i}\left(t_{j}\right)-r_{i}^{\text{ref}}\right|}^{2}\right]}^{1/2},$$ (9)

where T is the time over which one wants to average, and **ref** is the reference position of particle i. This reference position will be the time-averaged position of the same particle i.

The difference between RMSD and RMSF is that the latter is averaged over time, giving a value for each particle i, while for the RMSD the average is taken over the particles, giving time-specific values (Loschwitz et al., 2023, p. 2).

#### Hbonds, RoG, and SASA

Hydrogen bond analysis is the examination of interactions between a protein and a ligand within a 3.5-angstrom cutoff distance (da Fonseca et al., 2023; Yekeen et al., 2023). This analysis involves the identification of hydrogen bonds, which are crucial for the structural stability and molecular recognition within proteins. The core structural elements of proteins, such as α-helices and β-sheets, are stabilized by hydrogen bonds formed between the main chain carbonyl oxygen and amide nitrogen. These hydrogen bonds are responsible for conferring structural rigidity and specificity to intermolecular interactions. In this analysis, hydrogen bonds are defined as interactions in which an electronegative atom (acceptor A) interacts with a hydrogen atom covalently bonded to another electronegative atom (donor D). The criterion for a hydrogen bond entails less than 3.5 angstroms between the hydrogen atom (D) and the acceptor atom (A), as well as an angle (D-H-A) falling within the range of 150–210°. By this method, hydrogen bond interactions between the protein and ligand can be identified and characterized, revealing their significance in the context of molecular recognition and structural stability. RoG is an indicator of the compactness or overall size of a molecular structure. It is determined using the following formula (da Fonseca et al., 2023):

$$R_{g}={\left(\frac{\sum_{i}\;{\left|r_{i}\right|}^{2}m_{i}}{\sum_{i}\;m_{i}}\right)}^{1/2},$$ (10)

Where mi is the mass of atom i and **r**i the position of atom i concerning the center of mass of the molecule.

SASA is a measure that quantifies the surface area of a molecule or group of molecules that is accessible to a solvent, typically water. It is determined by defining a probe radius (often the van der Waals radius of water) and calculating the area that can be explored by this probe radius around the molecule or groups of interest while avoiding any overlap with atoms. Ultimately, SASA assesses the region of the molecule that is in contact with the solvent and can thus interact with other surrounding molecules.

#### MM/GBSA

Determining the binding strength between receptors and small ligands relies on assessing binding free energy. In this research, we employed the molecular mechanics/generalized Born surface area (MM/GBSA) method, utilizing the AMBER 14 software, to compute the binding free energy (Ylilauri and Pentikäinen, 2013; Kumari et al., 2014). The computation encompassed various components, including Δ_VDWAALS_, ΔE_EL_, ΔE_GB_, ΔE_SURF_, ΔG_GAS_, ΔG_SOLV_, and Δ_TOTAL_. The specific equations applied in this study for these calculations were outlined in a previous research publication (Faris et al., 2023c; Faris et al., 2023d).

## ADMET

To assess ADMET properties (Absorption, Distribution, Metabolism, Excretion, Toxicity) and drug-likeness, we utilized two specific web servers. The first, pkCSM (Pires et al., 2015; Azzam, 2023), was effectively employed to generate signatures encompassing five distinct pharmacokinetic property classes, facilitating the development of predictive models through regression and classification. Our findings demonstrate that pkCSM performs comparably or even superior to other freely available methods for various pharmacokinetic properties. The second server, SWISS-ADME (En-nahli et al., 2022; Azzam, 2023), served the purpose of computing physicochemical descriptors and predicting ADME parameters, pharmacokinetic properties, drug-like characteristics, and suitability for medicinal chemistry of one or multiple small molecules, providing valuable support for drug discovery.

## Results

### 2D-QSAR

#### MLR analysis

Among the objectives of the QSAR study is to understand the relationship between descriptors and their connection to biological activity. This enables the design of inhibitory molecules with favorable pharmaceutical characteristics and good stability. In this study, our target enzymes are JAK3 and JAK1, which are important for the autoimmune system. Based on a utilized dataset, we initially developed the MLR model. The model was derived utilizing the MLR methodology for studying the compounds with the pIC_50_ of JAK1 and JAK3, as illustrated by the following equations, respectively.

$$p{\text{IC}}_{50}\;\left(\text{JAK}1\right)=6.217\;–\;0.0025^{*}\text{ATS}7e+51.964^{*}\text{VC }–\;6+0.4343^{*}\text{nHssNH}+0.2803^{*}\text{minHBint}8,$$ (11)

where $R^{2}=0.95$ , $R^{2}\text{adj}=0.93$ , $R^{2}‐R^{2}\text{adj}=0.01$ , RMSE=0.13 , MAE=0.11 , s=0.15 , F=85.56

$$p{\text{IC}}_{50}\left(\text{JAK}3\right)=‐6.8736+0.0497^{*}\text{ATSC}7i+0.0874^{*}\text{VE}3_{\text{Dzp}}+4.5166*\text{SpMax}5_{\text{Bhp}}+0.0945+\text{slogPVSA}0,$$ (12)

where $R^{2}=0.91,$ R2adj=0.89 , $R^{2}‐R^{2}\text{adj}=0.01$ , RMSE=0.25 , MAE=0.11 s=0.28 , F=46.34,85.56

These models encompass both 4 crucial molecular descriptors for JAK1 and JAK3, which account for approximately 95% and 90% of the variations in pIC_50_, respectively (Figure 4). The observations indicate that all parameters (R^2^ (>0.6), MSE (a low value), F (a high value), and *p*-value (<0.05)) meet the statistically acceptable criteria. For the next, the results from internal and external validation will determine whether the models predict favorably or not.

### Interpretation of descriptors

The descriptors in the equations of models predicted have positive and negative coefficients, signifying their positive or negative contributions to the increase in pIC_50_ values. Molecules with specific features represented by these descriptors exhibit different inhibitory activities on JAK1 and JAK3. The coefficients and descriptor values can assist in designing molecules with targeted JAK inhibition as follows (Figure 5A):

Equation 1, which emphasizes the JAK1 series along with their corresponding pIC_50_ values: The negative coefficient (−0.0025) associated with ATS7e suggests that an increase in the value of ATS7e corresponds to a decrease in pIC_50_. This implies that molecules featuring specific characteristics represented by ATS7e may exhibit weaker inhibitory activity on JAK1. VC-6, on the other hand, has a positive coefficient (51.964). As VC-6 values increase, so does pIC_50_, indicating that molecules with higher VC-6 values tend to display higher inhibitory activity on JAK1. Regarding nHssNH, its positive coefficient (0.4343) suggests that molecules with a greater abundance of nHssNH are likely to have higher inhibitory activity on JAK1. Similarly, minHBint8, another molecular structure descriptor, has a positive coefficient (0.2803), indicating that molecules with more minHBint8 are prone to be more active on JAK1. For ATSC7i, the positive coefficient (0.0497) implies that an increase in the value of ATSC7i corresponds to a higher pIC_50_ for JAK3. This indicates that molecules with specific features represented by ATSC7i exhibit increased inhibitory activity on JAK3. Similarly, VE3_Dzp has a positive coefficient (0.0874), suggesting that molecules with higher VE3_Dzp values are more active on JAK3. As for SpMax5_Bhp, it demonstrates a significantly positive coefficient (4.5166), signifying those molecules with features represented by SpMax5_Bhp display notably higher inhibitory activity on JAK3. Additionally, slogPVSA0, with a positive coefficient of 0.0945, suggests that molecules with higher values of slogPVSA0 are more active on JAK3.

The Correlation Matrix for JAK1 provides the following insights: ATS7e and VC-6 display minimal correlations with the other descriptors. In contrast, nHssNH demonstrates a moderately positive correlation with minHBint8, implying a distinct relationship between these two descriptors. Notably, MinHBint8 exhibits a moderately negative correlation with nHssNH. The matrix predominantly underscores the presence of weak correlations, except for the moderate association between nHssNH and minHBint8 (Table 2).

**TABLE 2:** Correlation matrix of descriptors for JAK1 and JAK3.

Descriptor of JAK1	ATS7e	VC-6	nHssNH	minHBint8
ATS7e	1			
VC-6	0.065582	1		
nHssNH	0.421076	0.230613	1	
minHBint8	0.075777	−0.05149	−0.31699	1
Descriptor of JAK3	ATSC7i	VE3_Dzp	SpMax5_Bhp	slogPVSA0
ATSC7i	1			
VE3_Dzp	0.15599	1		
SpMax5_Bhp	−0.21115	−0.34598	1	
slogPVSA0	−0.38598	−0.36545	0.577294	1

In the Correlation Matrix for JAK3, we observe that ATSC7i and VE3_Dzp showcase weak correlations with the remaining descriptors. SpMax5_Bhp reveals limited correlations with the other descriptors, except for a moderately negative correlation with slogPVSA0. Intriguingly, SlogPVSA0 demonstrates moderately negative correlations with VE3_Dzp and SpMax5_Bhp, along with a moderately positive correlation with nHssNH. Overall, the matrix predominantly emphasizes weak correlations, with notable exceptions being the mentioned moderate relationships.

The correlation matrices for JAK1 and JAK3 primarily emphasize the existence of weak correlations among the descriptors, except for a few specific moderate associations. This underscores that these descriptors offer complementary information for predicting the activities of these targets, even in the presence of limited inter-descriptor correlations (Table 3).

**TABLE 3:** Descriptors for molecular structure analysis.

Descriptor	Type	Description
VC-6 Valence cluster, order 6	ChiClusterDescriptor	Measures the valence clustering tendency of atoms in a molecular structure
ATS7e	AutocorrelationDescriptor	Broto-Moreau autocorrelation of the topological structure at a lag of 7
ATSC7i		Centered Broto-Moreau autocorrelation at a lag of 7, weighted by first ionization potential and atomic Sanderson electronegativities
minHBint8	ElectrotopologicalStateAtom	Minimum E-State descriptors of the strength for potential Hydrogen Bonds of path length 8
TypeDescriptor nHssNH		Count of atom-type H E-State: NH-
VE3_Dzp	BaryszMatrixDescriptor	Logarithmic coefficients sum of the last eigenvector from the Barysz matrix, weighted by polarizabilities
Descriptor SpMax5_Bhp	BurdenModifiedEigenvalues	The largest absolute eigenvalue of the Burden modified matrix for n 5, weighted by relative polarizabilities
slogPVSA0	-	LogP value based on the Crippen method

### ANN model

In the search for a model capable of reliably predicting pIC_50_, we proceeded to develop the ANN model. The advantages of using ANN in QSAR include handling non-linear and complex data, automatic extraction of features from raw data, reduced experimental bias, flexibility with diverse data types, robustness against noise and outliers, the ability to predict various biological activities, easy interpretation through contribution analysis algorithms, and simple maintenance with the ability to update the model with new data.

SAR models developed using the ANN method exhibit high R^2^ (R^2^ = 0.99) and MSE (MSE = 0.03) values for JAK1, as well as high R^2^ (R^2^ = 0.97) and MSE (MSE = 0.05) values, like previous models. Additionally, they demonstrate an R^2^cv value (R^2^cv = 0.85) for JAK1 and R^2^cv value (R^2^cv = 0.80) for JAK3. These results indicate that the QSAR model employing the ANN approach can be utilized to predict the biological activity of the studied compounds. As depicted in Figure 5A, the distribution of pIC_50_ values for the studied compounds was highly similar in both datasets. This implies that the ANN models can predict pIC_50_ values that closely correspond to experimental values.

### Interpretation of descriptors

The importance of understanding the descriptor description is crucial for developing compound designs or identifying, judging, and better comprehending the relationship between these descriptors and activity. It also involves evaluating the augmentation or diminution of their reactivity.

The results in Supplementary Table S1 are associated with the internal validation of 2D-QSAR models for molecules targeting JAK1 and JAK3. Q^2 (^loo), indicating predictive quality, is better when closer to 1, with values around 0.9 (JAK1) and 0.8 (JAK3) signifying strong predictive capacity. R^2^-Q^2^loo measures observed variance, with low values like 0.0301 (JAK1) or 0.0801 (JAK3) indicating limited variance explained. RMSE and MAE estimate average prediction error, with lower values indicating higher model precision, although RMSE is lower for JAK1. PRESS quantifies prediction error in cross-validation, desiring lower values. CCC gauges the correlation between predicted and observed values, favoring values close to 1. JAK1 models show superior predictive performance over JAK3, as per Q^2 (^loo) and RMSE criteria.

According to external validation for the 2D-QSAR models for molecules targeting JAK1 and JAK3, the results presented in Supplementary Table S2, showing a model performance with Q^2^ values exceeding 0.5, indicate strong predictive capabilities of the models.

DoA refers to the chemical/property space covered by compounds in the training set used to build the QSAR model. It is important for a model to make predictions only within its defined DoA to ensure reliability. Compounds outside the DoA may lead to extrapolation errors. Y-randomization tests whether a model can discover true correlations or build random models. It involves randomly permuting biological activity values and checking if the permuted model has equal or better predictive power. A test set disjoint from the training set allows for an objective assessment of a model’s predictive ability within its DoA, considering overfitting. Both external validation (test set) and internal validation (e.g., leave-one-out/cross-validation) are recommended to properly evaluate a QSAR model’s performance. Ensuring the representativity and structural diversity of compounds in the training/test sets helps define the realistic boundaries of a model’s DoA. Proper DoA description and test set validation are essential to avoid overpredicted reliability and establish robust QSAR relationships.

## DoA analysis

The William plot depicting the AD of the models is presented in Figure 5B. The AD of the 2D-QSAR models was determined through leverage analysis, as represented by the Williams plot. The results obtained from the Williams plot demonstrate that all compound values in both the training and test sets were below the warning leverage threshold (h* = 0.625) for both JAK1 and JAK3 models. Sensitivity in the analysis of AD could be considered manageable or negligible for molecules outside of AD. A slight sensitivity or variability is demonstrated by the model with JAK1, with one outlier being detected in the domain of applicability. However, a more pronounced sensitivity is exhibited by the model with JAK3, with three outliers being observed in the domain of applicability. This suggests that a significant impact on the model’s performance in specific scenarios within the domain of applicability may be attributed to the presence of JAK3.

### Y-randomization

To minimize the potential occurrence of fortuitously selecting a strong association between molecular descriptors and pIC_50_, a Y-randomization methodology was executed. In this examination, a subset equivalent to twenty percent of the total compounds was randomly evaluated with a consistent set of twelve descriptors. Three measures (R^2^ r, R^2^_r_, cv, cRp^2^) were assessed and juxtaposed with the original model. As illustrated in Table 4, the values of the three measures for 10 randomly generated models were lower than those of the original model, signifying the robustness of the original model. In simpler terms, the original model did not exhibit any incidental correlation between molecular descriptors and biological activity.

**TABLE 4:** Y-randomization for each model with JAK1 and JAK3 activities.

Model/JAK1	R	R^2^	Q^2^	Random models parameters		
Original	0.94	0.88	0.33	Average r		0.54
Random 1	0.35	0.12	−0.93	Average r^2^		0.31
Random 2	0.58	0.33	−0.51	Average Q^2^		−0.81
Random 3	0.46	0.21	−0.73	cRp^2^		0.72
Random 4	0.35	0.12	−0.58			
Random 5	0.70	0.49	−0.77			
Random 6	0.60	0.36	−0.76			
Random 7	0.64	0.40	−1.12			
Random 8	0.44	0.19	−1.55			
Random 9	0.60	0.36	−0.28			
Random 10	0.74	0.55	−0.87			
Model/JAK3	R	R^2^	Q^2^	Random Models Parameters		
Original	0.87	0.76	0.46	Average r	0.58	
Random 1	0.61	0.38	−0.72	Average r^2^	0.35	
Random 2	0.64	0.41	−0.20	Average Q^2^	−0.40	
Random 3	0.35	0.12	−0.55	cRp^2^	0.57	
Random 4	0.42	0.18	−0.57			
Random 5	0.71	0.50	−0.02			
Random 6	0.60	0.36	−0.67			
Random 7	0.61	0.37	−0.63			
Random 8	0.65	0.42	−0.22			
Random 9	0.58	0.34	−0.33			
Random 10	0.630479	0.397504	−0.10			

### Pharmacophore hypothesis analysis

Ligand-based technologies, such as 3D-pharmacophore modeling, offer rapid screening of extensive compound databases, making them valuable for quick assessments. Conversely, structure-based approaches have the potential to generate a wider range of active compounds and provide significant insights into the target.

All the compounds selected from the database were employed to formulate a pharmacophoric hypothesis, delineating the essential molecular features necessary for binding with a receptor. From a pool of different pharmacophore hypotheses, we meticulously assessed and identified the most promising ones, leveraging various scoring criteria as elucidated in Supplementary Table S3, 4. Notably, we ascertained that the ADRRR hypothesis for JAK1 and the ADHRR hypothesis for JAK3, both of which are detailed in Supplementary Table S3, 4, emerged as the superior candidates among the hypotheses generated using the PHASE module. This determination was predicated on a comprehensive scoring function encompassing multiple parameters enumerated in the tables. The survival scoring function, crucial in the discernment of distinctive features within the proposed models and the ranking of these hypotheses, incorporated considerations such as selectivity, the number of ligands matched, relative conformational energy, and activity. Nonetheless, the models must possess the capability to distinguish between inactive and active molecules. If an inactive molecule garners a favorable score, it casts doubt on the validity of the hypothesis, as it fails to effectively discriminate between active and inactive compounds. Considering this, we introduced an adjusted survival score, which is computed by subtracting the score attributed to inactive molecules from the overall survival score. The two models, ADRRR_1 for the JAK1 target and ADHRR_1 for the JAK3 target are tools for predicting the activity of molecules with these targets (Supplementary Table S3, 4). The scores of these models, particularly the Survival Score, are critical measures of their ability to accurately predict the activity of molecules. For the selection of models for each target based solely on the Survival Score: JAK1 target: Model ADRRR_1 with a Survival Score of 5.68. JAK3 target: Model ADHRR_1 with a Survival Score of 5.73. These choices are based on the highest Survival Scores for each target, implying a better capability of these models to predict the activity of molecules concerning their respective targets.

**FIGURE 6:**
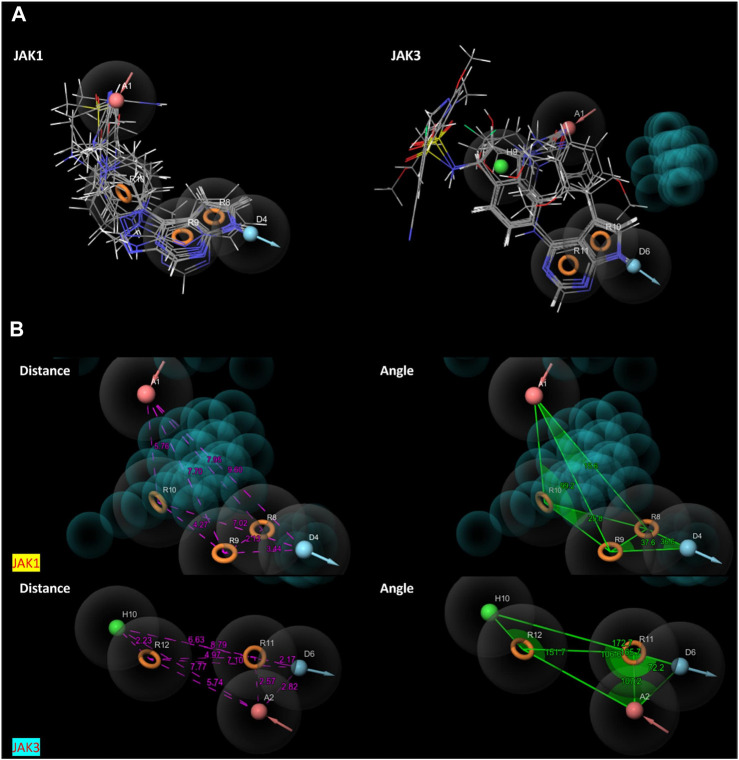
**(A)** Pharmacophore-based Ligand Alignment for JAK1 and JAK3 Inhibition Hypothesis. **(B)** The distances and angles between the features ADRRR_1 and ADHRR_1.

The characteristic features of each model for identifying inhibitor molecules of JAK1 and JAK3 are as follows: ADRRR_1, presents an acceptor and a donor, along with three aromatic rings. For ADHRR_1, it features an acceptor, a donor, hydrophobic properties, and two aromatic rings. Figure 6 depict the alignment of active compounds for each target, including angles and respective distances.

### Pharmacophore hypothesis validation

Two pharmacophore models, ADRRR and ADHRR, were assessed for their ability to discriminate between active compounds and decoys (See the supplementary file for more details). For ADRRR_1, the study encompassed 22 active compounds and a total of 28 ligands, including actives and decoys. The model displayed promising performance with a perfect BEDROC score of 1 at alpha = 160.9, indicating ideal active compound ranking. Additionally, the ROC value of 0.83 illustrated the model’s strong discriminatory power, while a Relative Enrichment Index (RIE) of 1.27 highlighted its capacity to effectively enrich activities. The area under the accumulation curve was 0.57, reflecting the model’s proficiency in prioritizing activities. In the top 20% of decoy results, a notable 31.8% of actives were successfully retrieved, further underscoring the model’s efficacy. Enrichment factors (EF) above 1.3 signified substantial enrichment, and the list of ranked actives was provided for comprehensive analysis. For ADHRR_1, the study involved 16 active compounds and 24 ligands in total. Like ADRRR_1, ADHRR_1 achieved a perfect BEDROC score of 1 at alpha = 160.9, indicating an exceptional ranking of active compounds. The ROC value of 0.75 demonstrated the model’s relatively strong ability to differentiate between active compounds and decoys. An RIE value of 1.34 highlighted the model’s effective enrichment of actives. The area under the accumulation curve, at 0.58, indicated the model’s proficiency in prioritizing activities. In the top 20% of decoy results, 12.5% of the actives were successfully recovered. Enrichment factors exceeding 1 suggested significant enrichment. Additionally, the model provided a list of ranked actives, facilitating in-depth analysis and experimental validation.

**FIGURE 7:**
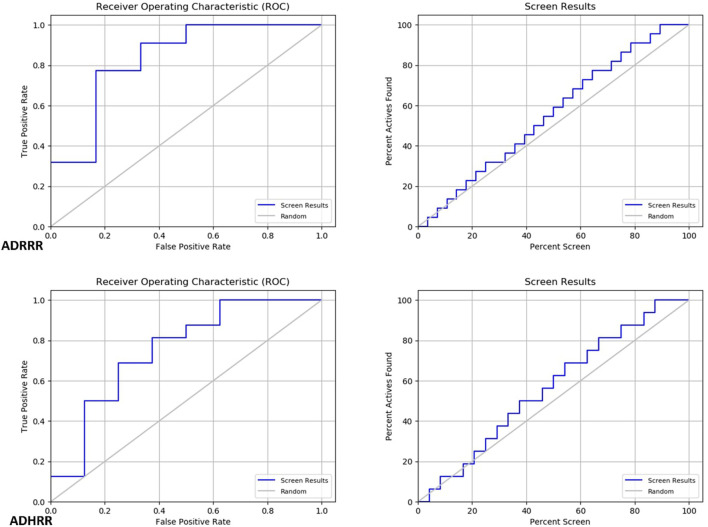
Roc and Screen results plots.

The pharmacophore models both exhibit strong performance in distinguishing between active compounds and decoys. They showcase high BEDROC scores, robust ROC values, and impressive enrichment factors, underscoring their efficacy in ranking active compounds. The ROC and Screen results in Figure 7 illustrate the trend of these models to predict with high accuracy the molecules with inhibitory activity, as observed in the screening to identify Baricitinib and Ruxolitinib Drugs among recent drug discoveries as JAK inhibitors. Furthermore, the models yield lists of ranked actives, which are valuable for subsequent analysis and experimental validation. These models demonstrate promising capabilities in identifying potential active compounds.

### Screening of database

After the validation of the pharmacophore models, the ADRRR_1 models were employed to identify selective inhibitors against JAK1 and ADHRR_1 that are selective against JAK3. This was achieved through screening a database containing JAK3 inhibitors, both of synthetic and natural origin, to predict their presence in the ZINCdata site as potential drug candidates. Table 5 showcases the results of compounds displaying a notable similarity, as indicated by their low RMSD values. All the molecules obtained undergo a molecular docking process to eliminate those with low binding affinity, thereby enhancing strong inhibitory activity. The molecules selected for each target with high affinity are presented in Table 5. These molecules have a predicted biological activity (pIC_50_) through the utilization of developed QSAR models, as shown in Tables 6, 7.

**TABLE 5:** The new identifier compounds targeting JAK1 and JAK3 with JAKInhibitorDB affinity.

Database type	ID	Smile	Affinity (Kcal/mol)
Target	**JAK1**		
JAKInhibitorDB	**ZINC66252131**	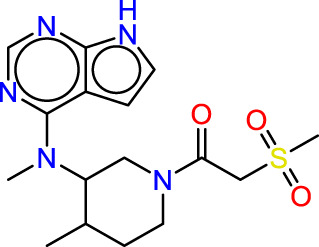	**−7.92**
	**ZINC13974878**	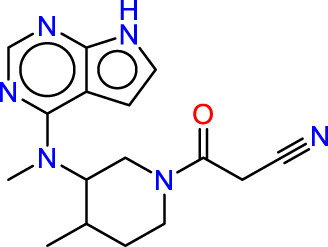	**−7.48**
	**ZINC261104647**	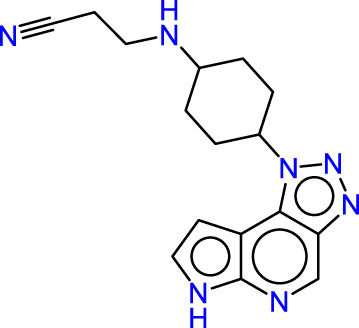	**−7.77**
	**ZINC96271468**	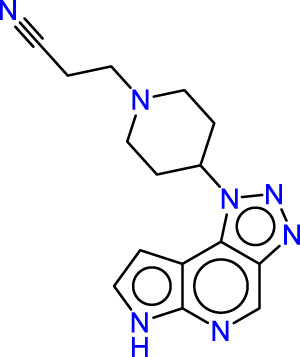	**−7.63**
	**ZINC45288940**	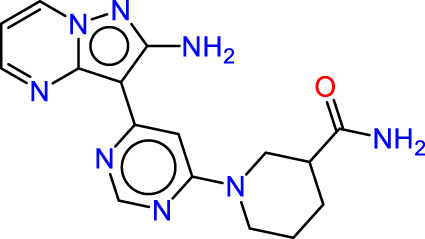	**−7.35**
	**ZINC66252348**	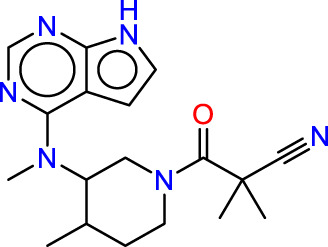	**−8.05**
Target	**JAK3**	**mol**	**S**
JAKInhibitorDB	**Ruxolitinib**	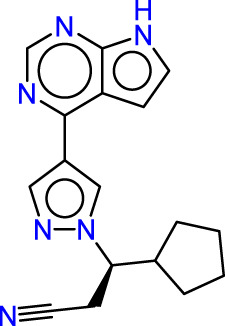	**−5.76**
	**Baricitinib**	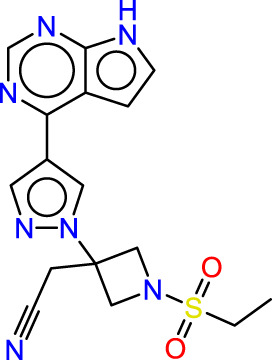	**−6.76**
	**ZINC96269459**	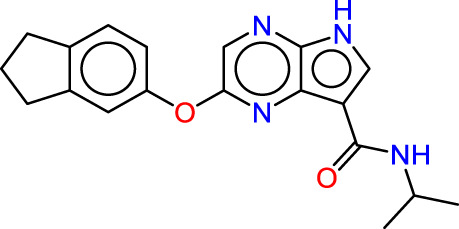	**−6.65**
	**ZINC95576632**	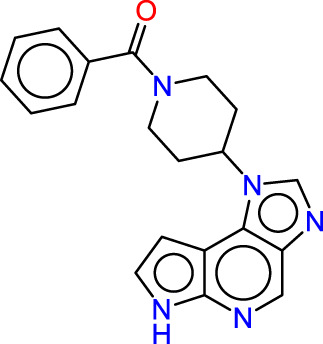	**−8.23**
	**ZINC101537469**	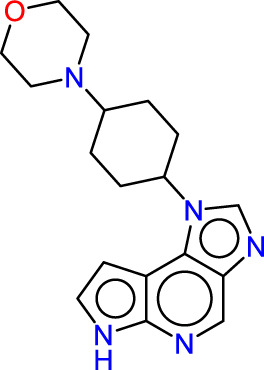	**−5.87**
Target	**JAK1**		
NaturalProductDB	**ZINC79189223**	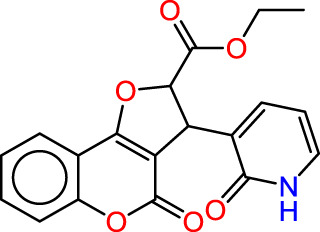	**−7.61**
	**ZINC40413912**	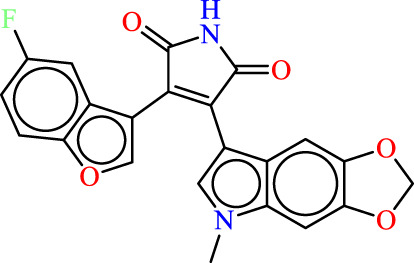	**−6.90**
	**ZINC38818041**	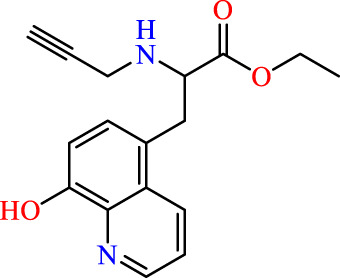	**−7.51**
Target	**JAK3**		
NaturalProductDB	**ZINC3843186**	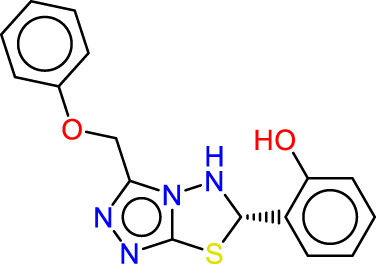	**−7.02**
	**ZINC95579616**	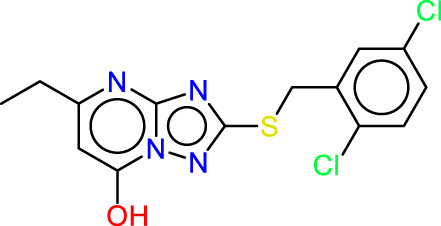	**−6.89**
	**ZINC3839141**	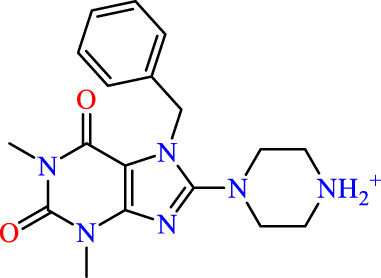	**−6.99**
	**Tofacitinib-Drug**	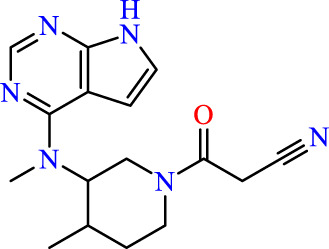	**−7.50**
Target			
JAK1	**A1**	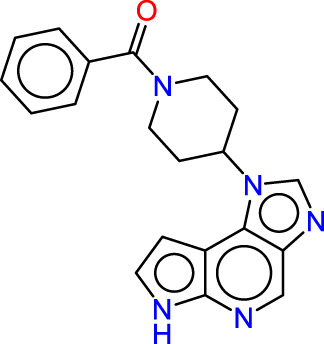	**−5.31**
JAK3	**A2**	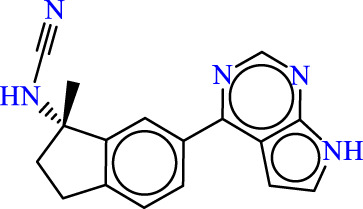	**−9.26**

Bold values are indicators based on ZINC data that were known from the first term ZINC.

**TABLE 6:** Molecules selected with the best affinity binding (Kcal/mol).

ZINC3843186 (JAK3)/pIC_50_ (pred) = 7.07	ZINC79189223 (JAK1)/pIC_50_ (pred) = 7.60
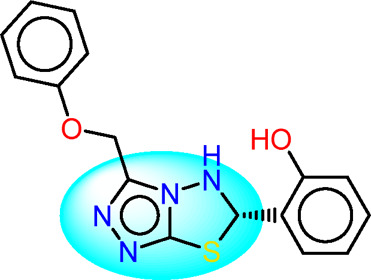	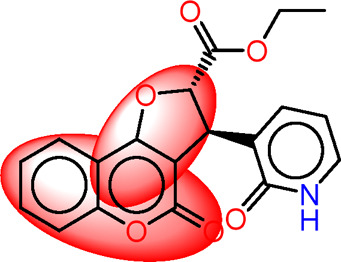
ZINC66252348 (JAK3)/pIC_50_ (pred) = 7.93	ZINC66252131 (JAK1)/pIC_50_ (pred) = 7.62
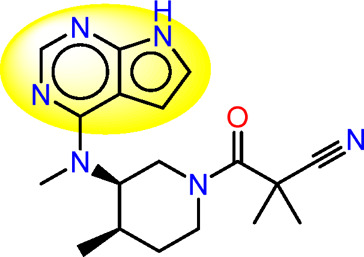	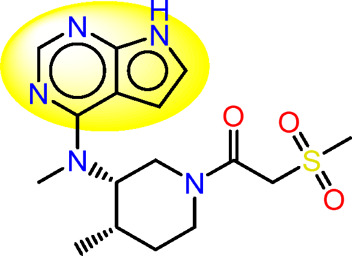
ZINC73069247 (JAK3) (Baricitinib)/pIC_50_ (pred) = 7.79	ZINC95576632 (JAK3)/pIC_50_ (pred) = 5.30
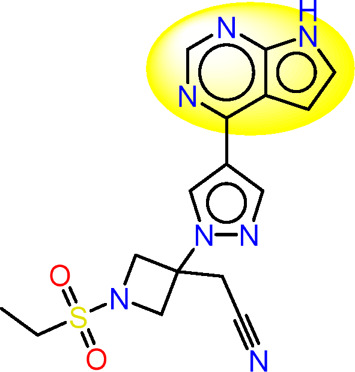	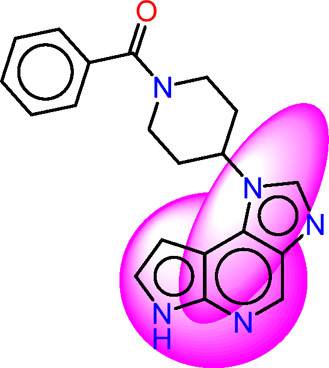
A1/pIC_50_ (pred) = 7.42	A2/pIC_50_ (pred) = 7.94
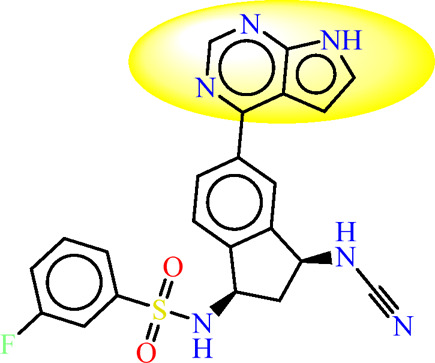	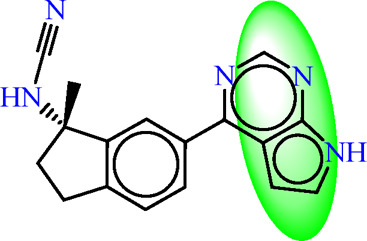

**TABLE 7:** MM/GBSA energy components for selected compounds.

MM/GBSA	ZINC3843186	ZINC79189223	ZINC66252348	ZINC66252131	Baricitinib	ZINC95576632	Tofacitinib
Δ_VDWAALS_	−37.44	−33.42	−37	−35.3	−43.9	−46.35	−22.82
ΔE_EL_	−30	−15.5	−25.01	−36.1	−25.2	−29.68	−32.93
ΔE_GB_	43.19	28.4	41.36	57.25	45	54	55.89
ΔE_SURF_	−4.97	−4.63	−5.09	−5.26	−5.44	−5.88	−3.34
ΔG_GAS_	−67.44	−48.92	−62.01	−71.39	−69.1	−76.03	−55.75
ΔG_SOLV_	38.22	23.77	36.27	52	39.56	48.13	52.55
Δ_TOTAL_	−29.22	−25.15	−25.74	−19.4	−29.55	−27.9	−3.2

Molecular docking is a computer-aided drug design technique that evaluates how new molecules bind to a biological target for drug discovery.

**FIGURE 8:**
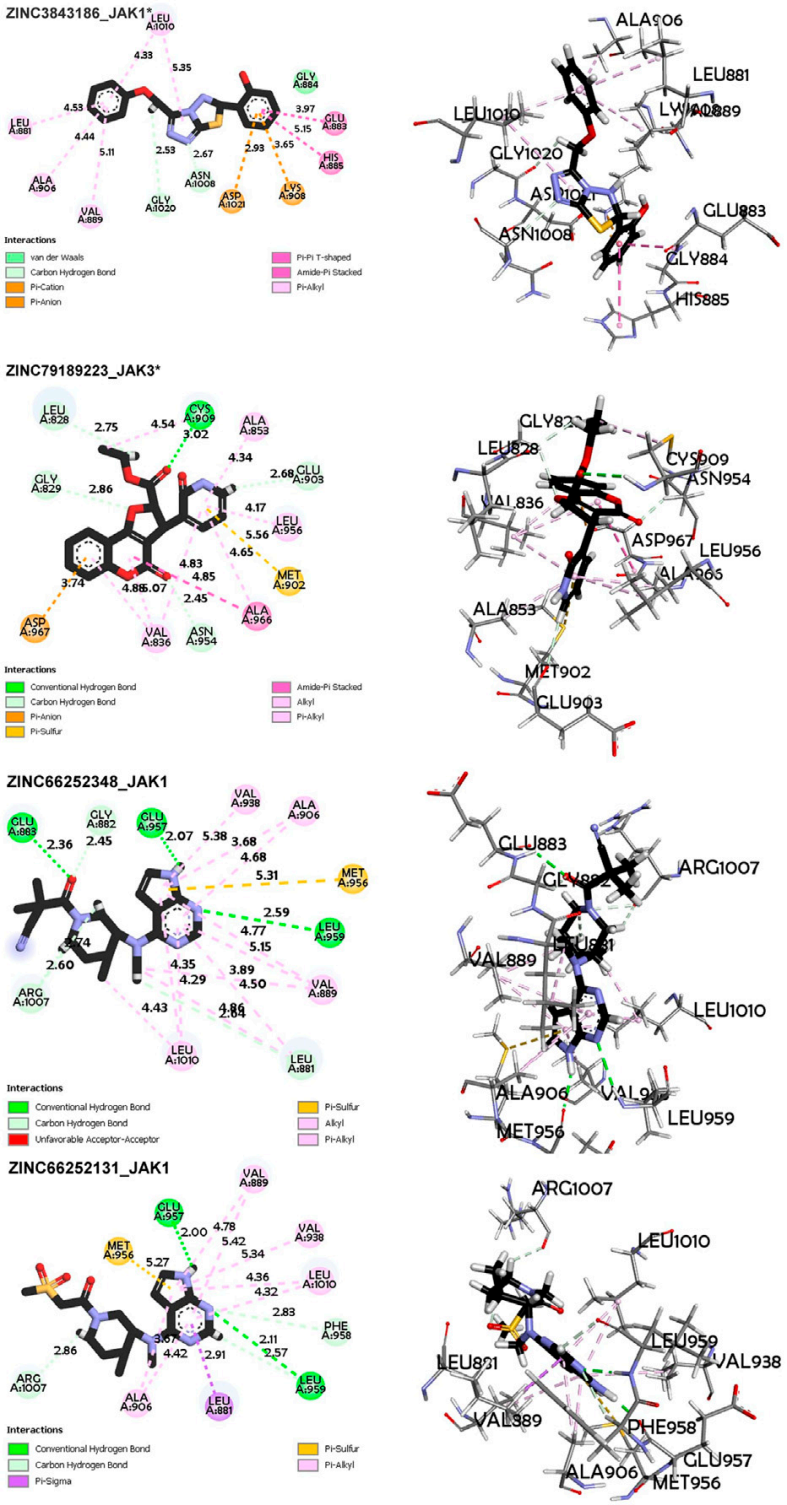
Non-covalent interaction analysis in 2D and 3D for compounds ZINC3843186, ZINC79189223, ZINC66252348 and ZINC66252131.

**FIGURE 9:**
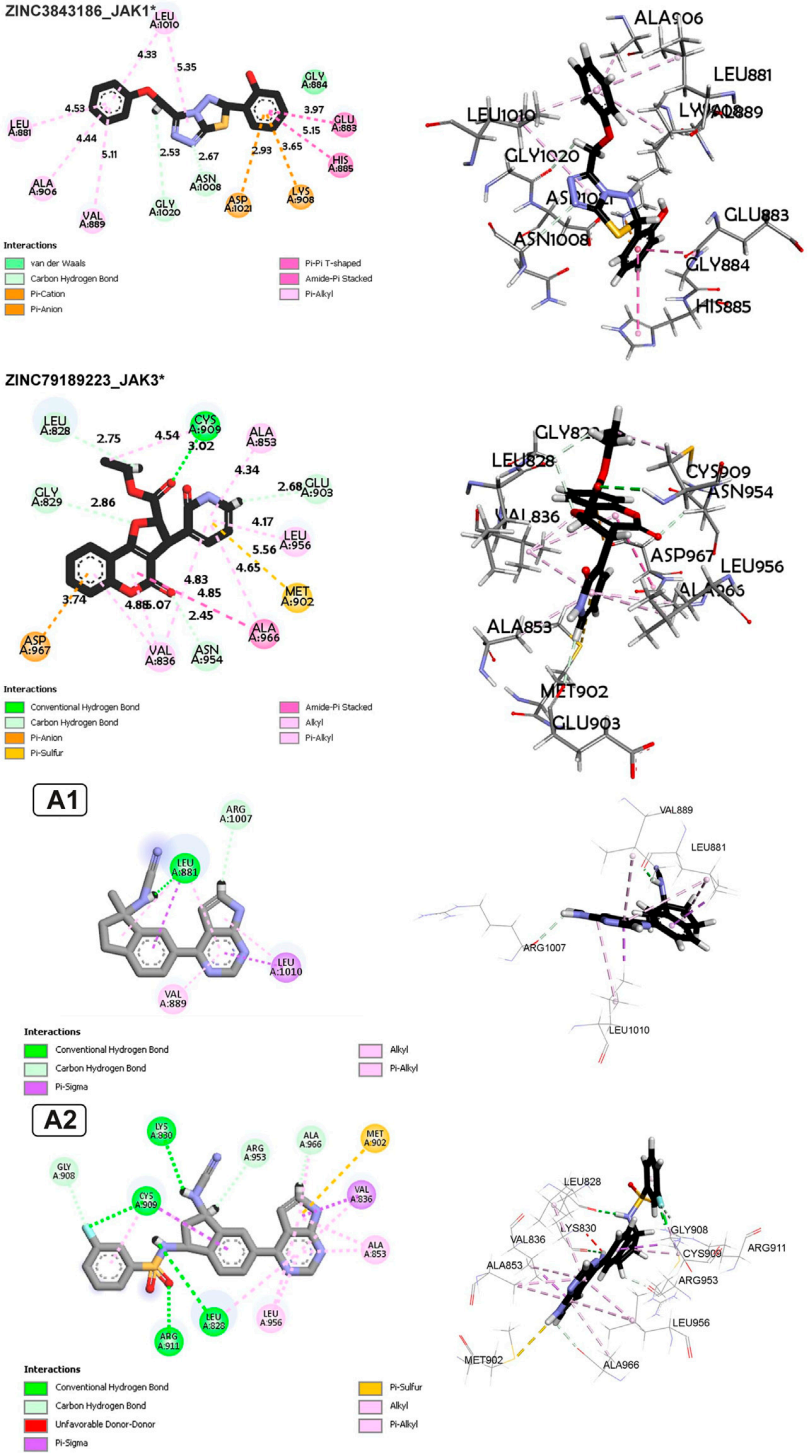
Non-covalent interaction analysis in 2D and 3D for compounds ZINC73069247 and ZINC95576632, A1, and A2.

Molecular docking analyses, as indicated in Supplementary Table S4 and illustrated in Figures 8, 9, demonstrate important non-covalent interactions expressing different affinities among the studied complexes.

Regarding the ZINC3843186_JAK1 complex (Figure 8), the main interactions observed are hydrogen bonds, particularly carbon-hydrogen bonds between the ligand and residues Gly1020 and Asn1008. This suggests that hydrogen bonds play a crucial role in ligand binding to the protein. An electrostatic Pi-Anion interaction is formed between Asp1021 and the ligand, indicating a specific electrostatic interaction. Another electrostatic interaction is observed in the form of a Pi-Cation bond between residue Lys908 and the ligand, suggesting a favorable electrostatic interaction. Hydrophobic interactions of the Amide-Pi Stacked, Pi-Alkyl, and Pi-Pi T-shaped types are also observed, indicating significant contributions of hydrophobic interactions to binding. The average distance between protein and ligand atoms ranges from 2.53 to 5.35 Å, indicating a range of distances for these interactions, with the minimum distance between residue Gly1020 and the ligand and the maximum distance between residue Leu1010 and the ligand. Additionally, for the ZINC79189223_JAK3 complex (Figure 8), the primary interactions consist of hydrogen bonds, including carbon-hydrogen bonds, suggesting a strong involvement of hydrogen bonds in ligand binding to the protein. There is also an electrostatic Pi-Anion interaction between Asp967 and the ligand, indicating an additional electrostatic interaction. Hydrophobic interactions of Pi-Alkyl, Pi-Sigma, Alkyl, and Amide-Pi Stacked types are also observed, suggesting significant contributions of hydrophobic interactions to binding. The average distance between protein and ligand atoms varies from 2.45 to 5.56 Å, indicating a range of distances for these interactions.

Similarly, for the ZINC66252348_JAK1 complex (Figure 9), the main interactions are hydrogen bonds, including carbon-hydrogen bonds, indicating that hydrogen bonds are the predominant interactions. Hydrophobic interactions of Pi-Alkyl, Alkyl, and Pi-Sigma types are also observed, contributing to ligand binding. The average distance between protein and ligand atoms varies from 2.07 to 5.37 Å, showing a range of distances for these interactions. For Complex ZINC66252131_JAK1 (Figure 9), the primary interactions are hydrogen bonds, including carbon-hydrogen bonds and Carbon Hydrogen Bond type, highlighting the importance of hydrogen bonds. A Pi-Sigma-type interaction is observed, indicating a specific electrostatic interaction. Pi-alkyl hydrophobic interactions are also present, strengthening the binding between the ligand and the protein. The average distance between protein and ligand atoms ranges from 1.99 to 5.42 Å.

For Complex ZINC73069247_JAK3 (Figure 9), the main interactions are hydrogen bonds, including carbon-hydrogen bonds, underscoring the importance of hydrogen bonds in ligand binding. There is an electrostatic Pi-Anion interaction between Asp967 and the ligand, indicating a specific electrostatic interaction. Hydrophobic interactions of Pi-Alkyl, Pi-Sigma, Amide-Pi Stacked, and Alkyl types are also observed, demonstrating the significance of hydrophobic interactions. The average vary between protein and ligand atoms varies from 2.67 to 5.38 Å, showing a range of distances for these interactions. For Complex ZINC79189223 _JAK3 (Figure 9), the primary interactions are hydrogen bonds, including carbon-hydrogen bonds, indicating the importance of hydrogen bonds in ligand binding. There is an electrostatic Pi-Anion interaction between Asp967 and the ligand, highlighting a specific electrostatic interaction. Pi-alkyl and Pi-Sulfur hydrophobic interactions are also observed, showing that hydrophobic interactions are a key factor in binding. The average distance between protein and ligand atoms ranges from 2.34 to 5.53 Å, indicating a range of distances for these interactions.

The newly designed compounds demonstrate non-covalent interactions in the following manner: In the case of compound A1 with JAK1, there is a hydrogen bond, along with pi-alkyl and pi-sigma interactions with Leu88, a pi-alkyl interaction with Val889, and a pi-sigma and alkyl interaction with Leu1010. Regarding compound A2 with JAK3, it features four hydrogen bonds with Cys909, Lys830, Arg911, and Leu828, as well as pi-alkyl and pi-sigma interactions with Cys909, a pi-alkyl interaction with Leu956, and another pi-alkyl interaction with Leu828. Additionally, there are two pi-alkyl interactions with Ala853, a hydrogen bond with Arg953 and Ala966, and a pi-sulfur interaction with Met902.

In addition, for the molecules in the series, we also consider molecular dynamics, selecting the molecules known for high activity in each series. For JAK1, A1 molecule 23 in Supplementary Table S1 with a pIC_50_ of 7.24 is chosen, and for JAK3, in Supplementary Table S1 A2, molecule 32 with a pIC_50_ of 7.96 is selected (Figure 9). These selections are crucial for understanding the molecular structures associated with high biological activity and guide our exploration of molecular interactions at the atomic level.

Molecular docking analysis revealed important non-covalent interactions between newly designed compounds and their biological targets. Hydrogen bonds, electrostatic interactions, and hydrophobic interactions were found to play significant roles in ligand binding. These findings provide valuable insights into the molecular structures and interactions underlying high biological activity, aiding in the development of potential drug candidates. Molecular dynamics simulations further supported the selection of promising molecules with high activity, enhancing our understanding of atomic-level interactions.

### The most active compounds in the studied series for JAK1 and JAK3

In the interaction between A1 and JAK1, hydrogen bonding takes center stage, with residues such as CYS909, ARG911, LEU828, LYS830, and others forming bonds at different distances. Notably, GLY908 engages in both carbon-hydrogen bonds and halogen interactions, demonstrating a multifaceted connection with the ligand. Furthermore, Pi-Sigma interactions involving VAL836 and CYS909 underscore the significance of pi interactions in the binding process. The A1-JAK1 complex exhibits Pi-Sulfur, Alkyl, and Pi-Alkyl interactions at various distances, highlighting the intricate nature of the binding interactions. In contrast, in the context of A2 with JAK3, hydrogen bonds form between LEU881 and ARG1007, emphasizing the importance of this interaction type. Pi-Sigma interactions involving LEU881 and LEU1010 contribute to the binding affinity at distances reflecting spatial proximity. Alkyl and Pi-Alkyl interactions, observed at varying distances with residues like LEU881, VAL889, and LEU1010, further enhance the stability of the A2-JAK3 complex.

**FIGURE 10:**
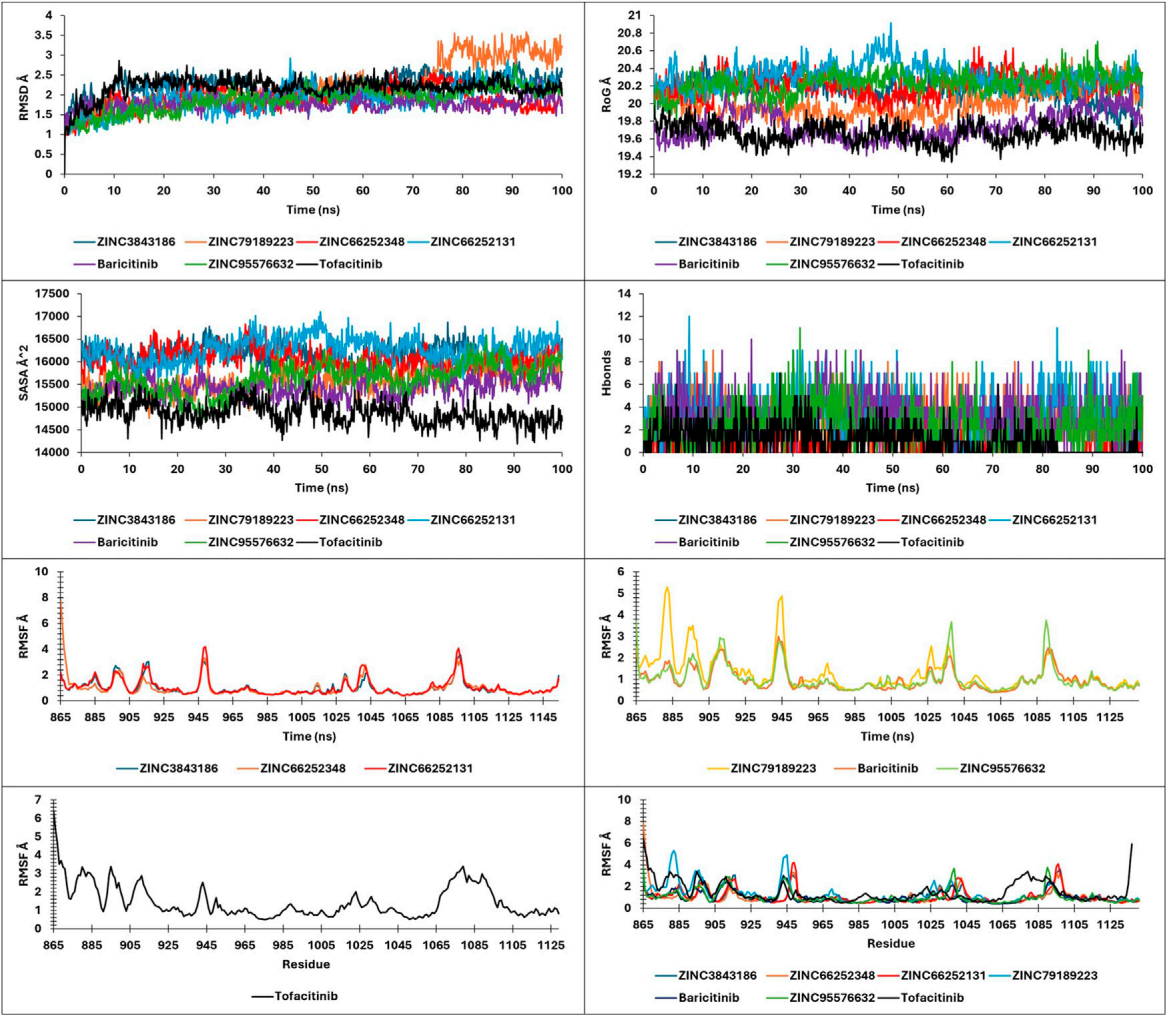
RMSD, RMSF, SASA, RoG, and Hbonds plots.

A note on the interactions between ZINC66252348 and ZINC73069247 (Baricitinib) in Figures 8, 9 with the target proteins shows the presence of unfavorable bonds, suggesting repulsive interactions that favor instability in the active pocket. However, despite the instability, their stability is maintained in the presence of hydrogen bonds, which play a crucial role in achieving remarkable stability with a significant affinity, as demonstrated by molecular dynamics (Figure 10).

The outcomes of molecular docking provide significant revelations about molecules with high affinity, emphasizing their close association with the quantity of formed bonds, particularly hydrogen bonds. Notably, compounds such as ZINC66252348, displaying an affinity of −8.05 kcal/mol, and A2 with −9.26 kcal/mol, showcase improved inhibitor binding facilitated by a greater number of hydrogen bonds. In contrast, A1, characterized by fewer hydrogen bonds and other bond types, exhibits a lower affinity of −5.31 kcal/mol, affirming that a heightened count of hydrogen bonds contributes to elevated affinity interactions. The docking analysis revealed important insights into the interactions of various complexes with JAK1 and JAK3 proteins. Hydrogen bonds were found to play a crucial role in ligand binding, along with electrostatic and hydrophobic interactions. The average distances between protein and ligand atoms varied, indicating the range of these interactions. Molecular dynamics simulations further supported the stability of selected compounds with high activity in each series. The analysis highlighted the significance of hydrogen bonding and other interactions in achieving strong affinity interactions. The new JAK1 and JAK3 inhibitor molecules exhibit high affinity and promising potential as drug candidates.

#### Molecular dynamics

To confirm the results of molecular docking, the molecules undergo an additional round of molecular docking and free-binding energy calculation. A thorough analysis of molecular dynamics simulation results is crucial for validating model stability and proper system equilibration. With Key metrics to examine, it is good practice to monitor these parameters at frequent intervals during long simulations to detect any deviations from expected behavior promptly (Smith et al., 2020). Molecular dynamics is a powerful technique for predicting favorable molecules for *in vitro* studies. By simulating molecular behavior, it provides insights into stability, solubility, target affinity, and interactions. Trajectory analysis identifies key conformations and interactions, guiding the selection of promising candidates for further investigation (Al-Karmalawy et al., 2023; Buskes et al., 2023).

The present study analyzed metrics including energy, pressure, temperature, drift extracted, RMSD, RMSF, SASA, RoG, and Hbonds from 100ns simulations, to validate the stability and convergence of different ligand-protein complexes (Figure 10; Figure 13).

### RMSD, RMSF, RoG, SASA, and hbonds analysis

Analysis of RMSD during a 100 ns simulation of the novel compounds revealed favorable stability ranging between 1 Å and 2.5 Å. All compounds exhibited a slight increase in RMSD during the initial 10 ns, followed by stability up to 100 ns, except for ZINC7918223, which experienced a 0.5 Å RMSD increase after 75 ns, indicating a pseudo-stability until 100 ns. For RoG and SASA analysis, the complexes displayed SASA values ranging from 14,000 to 16,500 Å^2^ and RoG values between 19.6 Å and 20.6 Å. As observed in the analysis of these parameters, the complexes tended to maintain their compactness throughout the trajectory. Hbonds analysis revealed that the compounds formed a minimum to a maximum range of 1–12 hydrogen bonds, which explains their high affinity. RMSF analysis provided insights into the residue stability of the studied complexes, highlighting the notable observation that the newly identified compounds exhibited greater residue stability compared to the tofacitinib drug. This indicates their inhibitory potential for both JAK1 and JAK3 in their respective environments.

The findings suggest that the novel compounds possess favorable stability, structural integrity, and hydrogen bonding patterns, making them promising candidates for further exploration as selective JAK1 and JAK3 inhibitors.

### Fel and PCA analysis

**FIGURE 11:**
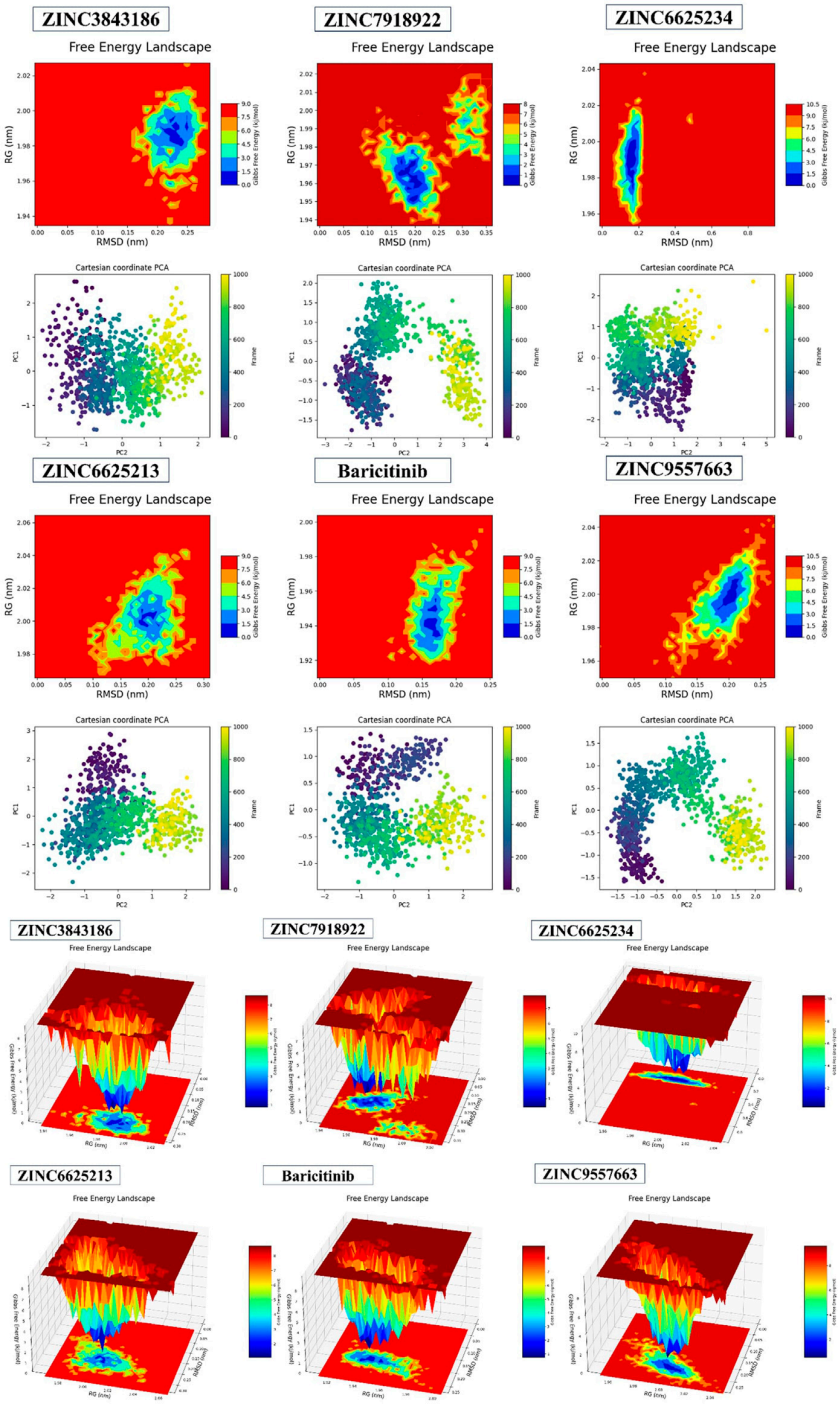
2D and 3DPlots of Fel and PCA.

**FIGURE 12:**
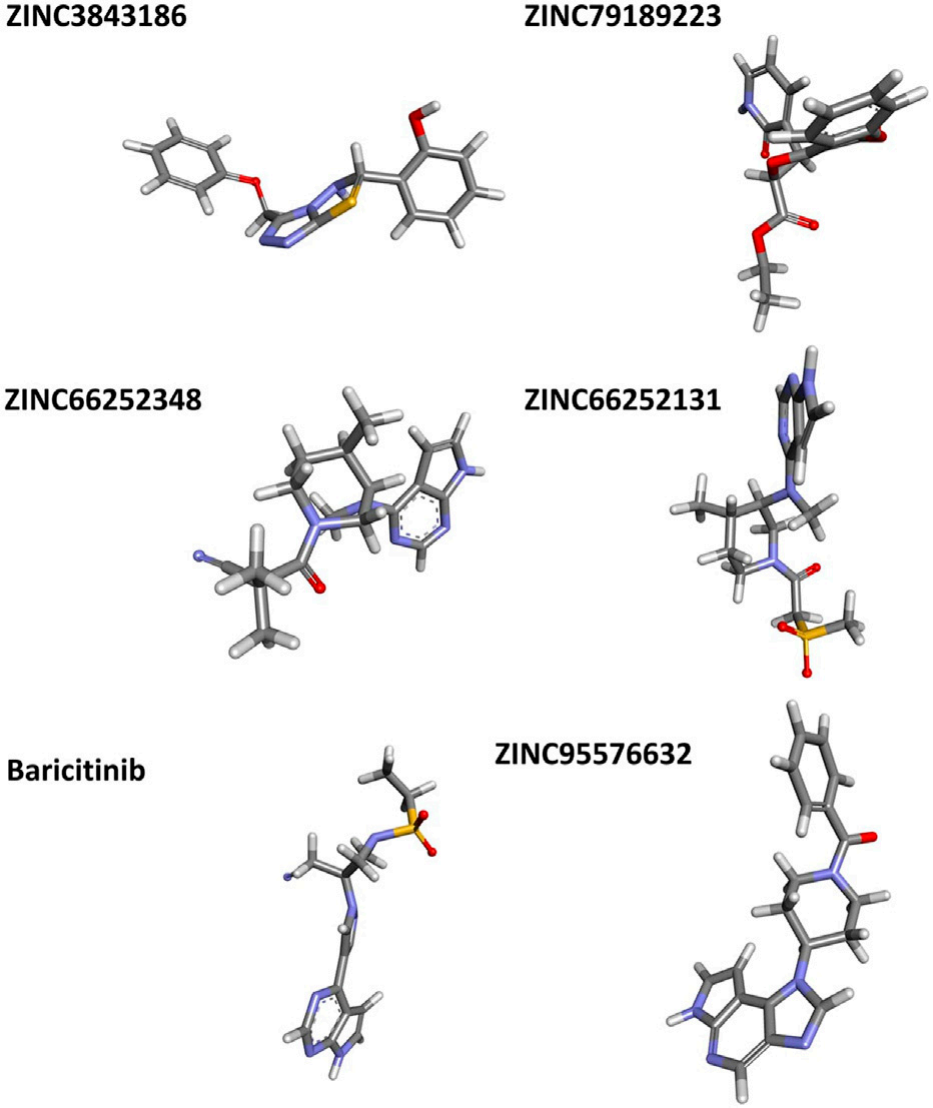
The most stable conformations were obtained using Fel and PCA.

The 2D results from Fel and PCA in Figures 11, 12 and the 3D results from Fel provide valuable insights. The Fel results suggest information that can be used to extract stable conformations during a 100ns simulation. PCA is employed to reduce data dimensionality by identifying the primary directions of variation, aiding in the visualization and comprehension of correlations or distinctions within the data. Specifically, for various compounds, the best minimum energy conformations are identified.

For ZINC3843186, this conformation is situated between 1.98 RG (nm) and 0.23 RMSD (nm), with PC1 ranging from −1 to 2 and PC2 from −2 to 2. ZINC79189223 features the best minima energy conformation located between 1.96 RG (nm) and 0.20 RMSD (nm), with PC1 spanning from −1.5 to 2 and PC2 from −3 to 4. ZINC66252348’s best minima energy conformation falls between 1.99 RG (nm) and 0.19 RMSD (nm), with PC1 ranging from −2 to 2 and PC2 from −2 to 5. As for ZINC66252131, the best minima energy conformation is found between 2.00 RG (nm) and 0.20 RMSD (nm), with PC1 between −2 and 3 and PC2 between −2 and 2. Baricitinib exhibits a best minima energy conformation located between 1.94 RG (nm) and 0.16 RMSD (nm). Lastly, ZINC95576632’s best minima energy conformation is situated between 2.00 RG (nm) and 0.20 RMSD (nm), with PC1 ranging from −1 to 1.5 and PC2 from −2 to 2. In conclusion, the results obtained from Fel and PCA offer insights into the stable conformations of various compounds during a 100ns simulation.

**FIGURE 13:**
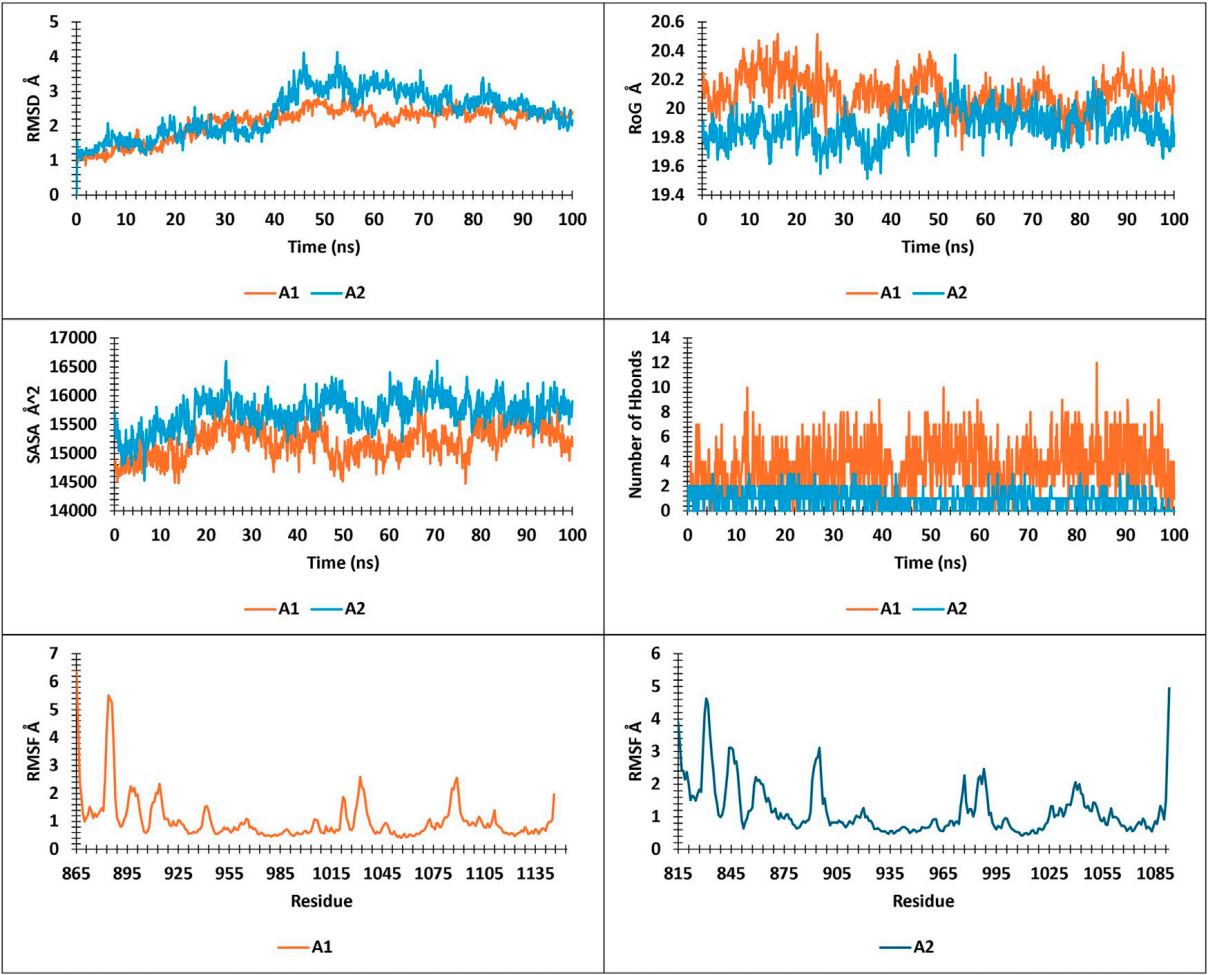
Rmsd, RMSF, SASA, and RoG analysis for **(A1, A2)**.

**FIGURE 14:**
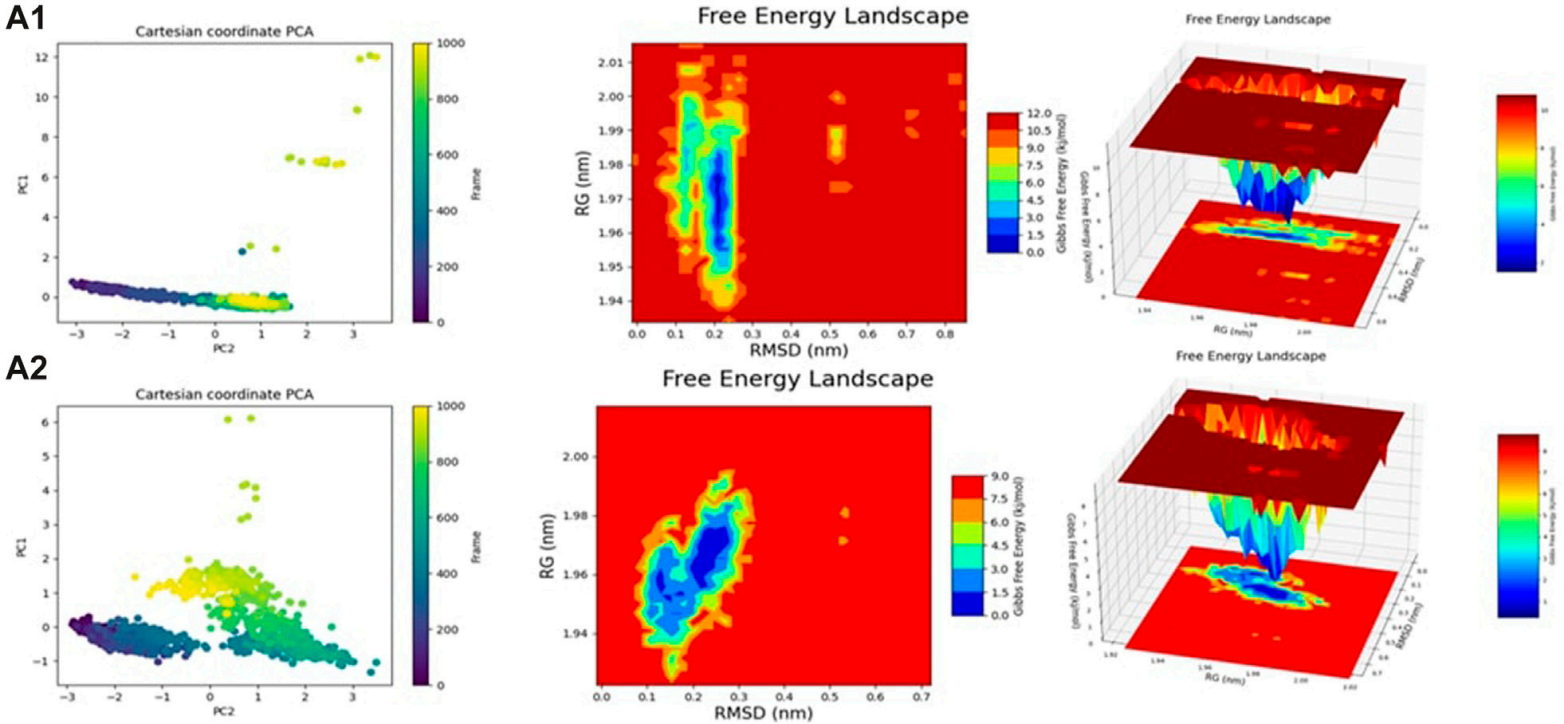
Fel and PCA analysis for **(A1, A2)**.

The comparison of the predicted New Composers with the compounds from the selected series identifies the best activity for each target of Series A1 (JAK1) and A2 (JAK3). Even on the analysis of molecular dynamics, it is observed that the new molecules show better stability, confirming the results of the previous analyses (Figures 13, 14).

#### MM-GBSA

The MM/GBSA results for the compound under study reveal significant insights. ΔV_DWAALS_ and ΔE_EL_ both exhibit negative values, signifying attractive van der Waals and electrostatic contributions to the total free energy, indicating favorable atom-environment interactions. Conversely, ΔE_GB_, positive in value, suggests that solvation in the solvent increases total free energy due to favorable interactions. Additionally, ΔE_SURF_ negative values imply that creating the solute’s surface in the solvent releases energy, possibly through better exposure to hydrophobic groups or reduced unfavorable solvent interactions. ΔG_GAS_, with negative values, indicates a preference for the gas phase due to lower free energy compared to the solvated phase. Meanwhile, ΔG_SOLV_, positive in value, represents increased free solvation energy, likely due to favorable solvent interactions.

Δ_TOTAL_ values indicate that compounds ZINC3843186, ZINC79189223, ZINC66252348, ZINC66252131, Baricitinib, and ZINC95576632 exhibit comparable or slightly lower total free energies than tofacitinib, implying similar or slightly reduced binding and stability properties.

### ADMET and drug-likness

Next, we assessed the similarity with drugs for the 8 potential compounds (Table 8). All compounds, except A2, adhered to the Veber, Lipinski, and Egan rules, indicating that these compounds were considered drug-like. However, A2 exhibited a violation due to a high weight violation: TPSA >131.6. Compounds 72 and 101 had a high molecular weight, violating the Veber, Lipinski, and Egan rules. The synthetic accessibility of the studied compounds ranged from 2 to 4.5, suggesting that these compounds can be synthesized as these values were closer to 1 (easy) rather than 10 (difficult).

**TABLE 8:** Drug-likeness assessment of compounds based on lipinski, Ghose, Veber, Egan, and Muegge rules, and synthetic accessibility.

Compounds	Lipinski #	Ghose #	Veber #	Egan #	Muegge #	Synthetic accessibility
ZINC3843186	Accept	Accept	Accept	Accept	Accept	3.75
ZINC79189223	Accept	Accept	Accept	Accept	Accept	4.24
ZINC66252348	Accept	Accept	Accept	Accept	Accept	3.48
ZINC66252131	Accept	Accept	Accept	Accept	Accept	3.51
Baricitinib	Accept	Accept	Accept	Accept	Accept	3.07
ZINC95576632	Accept	Accept	Accept	Accept	Accept	2.29
A1	Accept	Accept	Accept	Accept	Accept	2.88
A2	Accept	Accept	Accept	No	Accept	3.95

#violations

**FIGURE 15:**
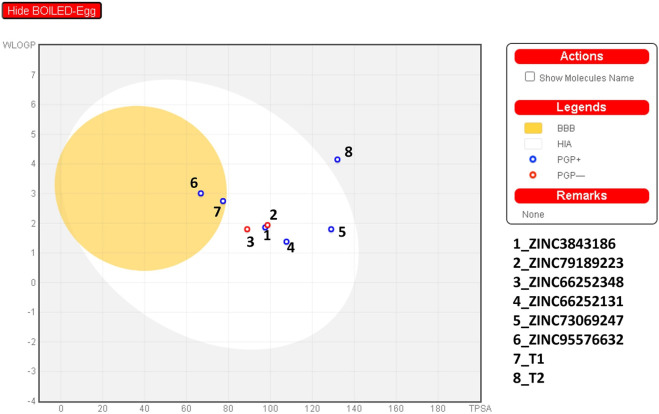
The BOILED-Egg for identifying the new compounds.

The optimal values of the studied compounds for polarity, lipophilicity, solubility, metabolism, size, saturation, and flexibility are also provided in Supplementary Table S3. Subsequently, we evaluated the ADMET of potential compounds while considering their similarity to drugs. As indicated in Table 3, all studied compounds exhibit moderate water solubility. Furthermore, all compounds demonstrate high absorption in the human intestine and permeability through CaCO2. All molecules possess P-glycoproteins, except for compounds ZINC79189223 and ZINC66252348. As depicted in Figure 15, these molecules do not cross the blood-brain barrier (BBB) for safety reasons and are also non-toxic. Based on these factors, and considering previous studies, we can select ZINC79189223 and ZINC66252348 as potential drugs that can serve as tools for the inhibition of JAK1 and JAK3.

#### Biological activity

Comparison of the predicted biological activities (Supplementary Table S6) of new drugs 2 and 3 with tofacitinib: Tofacitinib, mainly targets Janus kinases (JAK) 1, 2, 3 which are tyrosine kinases involved in interleukin signaling. Indicates anti-inflammatory, and immunosuppressive activities and in the treatment of autoimmune diseases such as rheumatoid arthritis. Compound 2: Has a more diverse range of activities targeting various metabolism enzymes such as cytochromes P450 and with vasodilatory, anti-vasoconstrictor effects. It also indicates anti-inflammatory and antipruritic activities. It is less specific than tofacitinib on JAK kinases. Compound 3, Mainly targets tyrosine kinases like tofacitinib but more broadly on JAK1-3 kinases, other kinases involved in cellular signaling. Indicates anti-inflammatory, immunosuppressive activities, in the treatment of autoimmune and rheumatic diseases like tofacitinib. It could have a broader activity than tofacitinib given the larger number of predicted kinase targets. Therefore, as a result, Compound 3 seems to have an activity profile closer and broader than tofacitinib, while Compound 2 has more varied activities also targeting enzymes other than kinases.

## Conclusion

In conclusion, our in-depth study aimed at discovering novel selective inhibitors against JAK1 and JAK3 has led to the identification of optimal compounds exhibiting both favorable affinity and stability during a 100 ns trajectory. The predictive models, specifically the 2D-QSAR MLR models developed by the ANN model, have demonstrated their capability to foresee biological activity and stability. These models can be further utilized in the design of new molecules for future studies. Similarly, the pharmacophore model aids in identifying key characteristics through the screening of basic JAK3 inhibitor molecules, guiding the identification of more potent species. This approach, coupled with Computer-Aided Drug Design (CADD), has revealed promising biological activity, exemplified by the compound ZINC79189223. Notably, this compound exhibits biological activity primarily against tyrosine kinases, resembling tofacitinib, with a broader impact on JAK1-3 kinases and other kinases involved in cellular signaling.

## Data Availability

The original contributions presented in the study are included in the article/Supplementary Material, further inquiries can be directed to the corresponding author.
