# Supplementary material for: Revealing innovative JAK1 and JAK3 inhibitors: a comprehensive study utilizing QSAR, 3D-Pharmacophore screening, molecular docking, molecular dynamics, and MM/GBSA analyses
Source: Front Mol Biosci. 2024 Mar 7;11:1348277. doi: 10.3389/fmolb.2024.1348277 (PMC10956358; doi:10.3389/fmolb.2024.1348277)
Supplement: Supplementary file 1 [file DataSheet1.zip › Supplementry File/Supplementry File.docx]

Table S1 Inhibitory Activity of Cyanamide-Based JAK1 and JAK3 Compounds: Conversion of IC50 to pIC50 Values.

| Compound number | SMILES | pIC_50_(JAK1) | pIC_50_(JAK3) |
| --- | --- | --- | --- |
| 1 | c1cc(cc(c1)S(=O)(=O)N[C@@H]2C[C@@H](c3c2ccc(c3)c4c5cc[nH]c5ncn4)NC#N)F | 5.57 | 7.97 |
| 2 | c1cc(cc(c1)S(=O)(=O)N[C@@H]2C[C@@H](c3c2ccc(c3)c4c5cc[nH]c5ncn4)NC#N)C#N | 5.77 | 7.86 |
| 3 | COc1ccc(cc1)S(=O)(=O)N[C@@H]2C[C@@H](c3c2ccc(c3)c4c5cc[nH]c5ncn4)NC#N | 5.76 | 7.8 |
| 4 | COCCc1c[nH]c2c1c(ncn2)c3cccc(c3)NC(=O)C=C | 4 | 7.51 |
| 5 | C[C@H]1CC[C@H](CN1C(=O)C=C)Nc2c3cc[nH]c3ncn2 | 4.01 | 7.48 |
| 6 | CS(=O)(=O)N[C@@H]1C[C@@H](c2c1ccc(c2)c3c4cc[nH]c4ncn3)NC#N | 5.72 | 7.42 |
| 7 | Cn1c(ccn1)S(=O)(=O)N[C@@H]2C[C@@H](c3c2ccc(c3)c4c5cc[nH]c5ncn4)NC#N | 5.4 | 7.42 |
| 8 | c1ccc(cc1)c2c[nH]c3c2c(ncn3)c4ccc5c(c4)[C@H](CC5)NC#N | 5.48 | 7.31 |
| 9 | COCCc1c[nH]c2c1c(ncn2)c3ccc4c(c3)N(CCO4)C(=O)C=C | 4 | 7.31 |
| 10 | C[C@@]1(CCc2c1cc(cc2)c3c4cc[nH]c4ncn3)NC#N | 7.24 | 7.25 |
| 11 | c1cc(cc(c1)c2c[nH]c3c2c(ncn3)c4ccc5c(c4)[C@H](CC5)NC#N)CO | 4.02 | 7.11 |
| 12 | C[C@]1(CCc2c1cc(cc2)c3c4cc[nH]c4ncn3)NC#N | 7.03 | 7.04 |
| 13 | COCCS(=O)(=O)N[C@@H]1C[C@@H](c2c1ccc(c2)c3c4cc[nH]c4ncn3)NC#N | 5.06 | 6.95 |
| 14 | c1cc2c(cc1c3c4cc[nH]c4ncn3)[C@H](C[C@H]2O)NC#N | 4.14 | 6.79 |
| 15 | c1cc2c(cc1c3c4cc[nH]c4ncn3)[C@H](CC2)NC#N | 6.19 | 6.59 |
| 16 | c1cc2c(cc1c3c4cc[nH]c4ncn3)[C@H]([C@@H](C2)O)NC#N | 5.9 | 6.55 |
| 17 | COCCc1c[nH]c2c1c(ncn2)c3ccc4c(c3)N(CC(O4)(F)F)C#N | 4 | 6.34 |
| 18 | C[C@H]1Cc2ccc(cc2[C@@H]1NC#N)c3c4cc[nH]c4ncn3 | 6.15 | 6.22 |
| 19 | c1cc2c(cc1c3c4cc[nH]c4ncn3)[C@H](CC2)NC#N | 6.04 | 6.15 |
| 20 | c1cc2c(cc1c3c4cc[nH]c4ncn3)[C@H](CCO2)NC#N | 5.92 | 6.11 |
| 21 | c1cc2c(cc1c3c4cc[nH]c4ncn3)[C@H](CCO2)NC#N | 5.93 | 5.92 |
| 22 | COCCc1c[nH]c2c1c(ncn2)c3ccc4c(c3)N(CCO4)C#N | 5.19 | 5.91 |
| 23 | c1cc2c(cc1c3c4cc[nH]c4ncn3)[C@H](C[C@@H]2O)NC#N | 5.96 | 5.87 |
| 24 | C[C@@H]1Cc2ccc(cc2[C@@H]1NC#N)c3c4cc[nH]c4ncn3 | 6.01 | 5.82 |
| 25 | COCCc1c[nH]c2c1c(ncn2)c3ccc4c(c3)[C@H](CC4)NC#N | 5.12 | 5.8 |
| 26 | c1cc2c(cc1c3c4cc[nH]c4ncn3)[C@@H](CC2)NC#N | 6.07 | 5.24 |
| 27 | COCCc1c[nH]c2c1c(ncn2)c3ccc4c(c3)[C@H](CCO4)NC#N | 5.42 | 5.23 |
| 28 | c1cc2c(cc1c3c4cc[nH]c4ncn3)[C@@H](CCO2)NC#N | 6.18 | 5.21 |
| 29 | CN(C#N)[C@H]1CCc2c1cc(cc2)c3c4cc[nH]c4ncn3 | 5.12 | 5 |

Table S2 Pharmacophore Models Performance Summary.

| **ADRRR** |
| --- |
| Enrichment Report  -----------------  Actives file: hvalidation_5-actives.txt  Results: hvalidation_5-ADRRR_1-hits.csv  Total actives: 22  Total ligands(actives+decoys): 28  Number of ranked actives: 22  BEDROC(alpha=160.9, alpha*Ra=126.4214): 1.000  BEDROC(alpha=20.0, alpha*Ra=15.7143): 0.997  BEDROC(alpha=8.0, alpha*Ra=6.2857): 0.958  ROC: 0.83  RIE: 1.27  Area under accumulation curve: 0.57  Ave. Number of outranking decoys: 1  Minimum Tc over all active pairs: n/a  Count and percentage of actives in top N% of decoy results.  % Decoys \| 1%\| 2%\| 5%\| 10%\| 20%\|  # Actives \| 0\| 0\| 0\| 0\| 7\|  % Actives \| 0.0\| 0.0\| 0.0\| 0.0\| 31.8\|  Count and percentage of actives in top N% of results.  % Results \| 1%\| 2%\| 5%\| 10%\| 20%\|  # Actives \| 0\| 0\| 1\| 2\| 5\|  % Actives \| 0.0\| 0.0\| 4.5\| 9.1\| 22.7\|  Enrichment Factors with respect to N% sample size.  % Sample \| 1%\| 2%\| 5%\| 10%\| 20%\|  EF \| n/a\| 1.3\| 1.3\| 1.3\| 1.3\|  EF* \| inf\| inf\| inf\| 1.9\| 1.9\|  EF' \| inf\| inf\| inf\| 1.9\| 1.9\|  DEF \| n/a\| n/a\| n/a\| n/a\| n/a\|  DEF* \| n/a\| n/a\| n/a\| n/a\| n/a\|  DEF' \| n/a\| n/a\| n/a\| n/a\| n/a\|  Eff \| -1\| -1\| -1\| -1\| 0.228\|    Enrichment Factors with respect to N% actives recovered.  % Actives \| 40%\| 50%\| 60%\| 70%\| 80%\| 90%\| 100%\|  EF \| 1.1\| 1.2\| 1.2\| 1.2\| 1.1\| 1.2\| 1.1\|  EF* \| 2.5\| 3\| 3.5\| 4.1\| 2.5\| 2.7\| 2\|  EF' \| 2.2\| 2.4\| 2.6\| 2.8\| 2.9\| 2.9\| 2.6\|  FOD \| 0.04\| 0.06\| 0.08\| 0.09\| 0.1\| 0.1\| 0.2\| |
| **ADHRRR** |
| Enrichment Report  Actives file: hvalidation_2-actives.txt  Results: hvalidation_2-ADHRR_3-hits.csv  Total actives: 16  Total ligands(actives+decoys): 24  Number of ranked actives: 16  BEDROC (alpha=160.9, alpha*Ra=107.2667): 1.000  BEDROC (alpha=20.0, alpha*Ra=13.3333): 0.893  BEDROC (alpha=8.0, alpha*Ra=5.3333): 0.826  ROC: 0.75  RIE: 1.34  Area under accumulation curve: 0.58  Ave. Number of outranking decoys: 2  Minimum Tc over all active pairs: n/a  Count and percentage of actives in top N% of decoy results.  % Decoys \| 1%\| 2%\| 5%\| 10%\| 20%\|  # Actives \| 0\| 0\| 0\| 0\| 2\|  % Actives \| 0.0\| 0.0\| 0.0\| 0.0\| 12.5\|  Count and percentage of actives in top N% of results.  % Results \| 1%\| 2%\| 5%\| 10%\| 20%\|  # Actives \| 0\| 0\| 1\| 2\| 3\|  % Actives \| 0.0\| 0.0\| 6.2\| 12.5\| 18.8\|    Enrichment Factors with respect to N% sample size.  % Sample \| 1%\| 2%\| 5%\| 10%\| 20%\|  EF \| n/a\| n/a\| 1.5\| 1.5\| 1.2\|  EF* \| inf\| inf\| inf\| 1\| 2\|  EF' \| inf\| inf\| inf\| 1\| 2\|  DEF \| n/a\| n/a\| n/a\| n/a\| n/a\|  DEF* \| n/a\| n/a\| n/a\| n/a\| n/a\|  DEF' \| n/a\| n/a\| n/a\| n/a\| n/a\|  Eff \| -1\| -1\| -1\| -1\| -0.231\|  Enrichment Factors with respect to N% actives recovered.  % Actives \| 40%\| 50%\| 60%\| 70%\| 80%\| 90%\| 100%\|  EF \| 1.3\| 1.3\| 1.3\| 1.2\| 1.2\| 1.1\| 1.1\|  EF* \| 3\| 4\| 2.5\| 2.8\| 2.2\| 1.8\| 1.6\|  EF' \| 1.7\| 2\| 2.1\| 2.1\| 2.1\| 2\| 1.9\|  FOD \| 0.08\| 0.09\| 0.1\| 0.1\| 0.2\| 0.2\| 0.2\| |

Table S3 Docking Molecular Analysis

| ZINC3843186_JAK1* | Distance | Category | Types | ZINC66252348_JAK1 | | Distance | Category | Types |
| --- | --- | --- | --- | --- | --- | --- | --- | --- |
| GLY1020 | 2.52986 | Hydrogen Bond | Carbon Hydrogen Bond | GLU957 | | 2.0706 | Hydrogen Bond | Hydrogen Bond |
| ASN1008 | 2.67082 | Hydrogen Bond | Carbon Hydrogen Bond | GLU883 | | 2.35814 | Hydrogen Bond | Hydrogen Bond |
| ASP1021 | 2.93495 | Electrostatic | Pi-Anion | GLY882 | | 2.45268 | Hydrogen Bond | Carbon Hydrogen Bond |
| LYS908 | 3.64756 | Electrostatic | Pi-Cation | LEU959 | | 2.59484 | Hydrogen Bond | Hydrogen Bond |
| GLU883 | 3.96507 | Hydrophobic | Amide-Pi Stacked | ARG1007 | | 2.60097 | Hydrogen Bond | Carbon Hydrogen Bond |
| LEU1010 | 4.33175 | Hydrophobic | Pi-Alkyl | LEU881 | | 2.63588 | Hydrogen Bond | Carbon Hydrogen Bond |
| ALA906 | 4.43548 | Hydrophobic | Pi-Alkyl | ARG1007 | | 2.74208 | Hydrogen Bond | Carbon Hydrogen Bond |
| LEU881 | 4.525 | Hydrophobic | Pi-Alkyl | ALA906 | | 3.67755 | Hydrophobic | Pi-Alkyl |
| VAL889 | 5.1063 | Hydrophobic | Pi-Alkyl | VAL889 | | 3.88568 | Hydrophobic | Alkyl |
| HIS885 | 5.15486 | Hydrophobic | Pi-Pi T-shaped | LEU1010 | | 4.28849 | Hydrophobic | Pi-Alkyl |
| LEU1010 | 5.34988 | Hydrophobic | Pi-Alkyl | LEU1010 | | 4.35377 | Hydrophobic | Pi-Alkyl |
|  | | | | | LEU1010 | 4.43349 | Hydrophobic | Alkyl |
|  |  |  |  |  | LEU881 | 4.49756 | Hydrophobic | Pi-Alkyl |
|  |  |  |  |  | ALA906 | 4.68442 | Hydrophobic | Pi-Alkyl |
|  |  |  |  |  | VAL889 | 4.76604 | Hydrophobic | Pi-Alkyl |
|  |  |  |  |  | LEU881 | 4.85903 | Hydrophobic | Alkyl |
|  |  |  |  |  | VAL889 | 5.14851 | Hydrophobic | Pi-Alkyl |
|  |  |  |  |  | MET956 | 5.30783 | Other | Pi-Sulfur |
|  |  |  |  |  | VAL938 | 5.37553 | Hydrophobic | Pi-Alkyl |
| ZINC79189223_JAK3* | Distance | Category | Types | ZINC66252131_JAK1 | | Distance | Category | Types |
| ASN954 | 2.44952 | Hydrogen Bond | Carbon Hydrogen Bond | GLU957 | | 1.99993 | Hydrogen Bond | Hydrogen Bond |
| GLU903 | 2.6817 | Hydrogen Bond | Carbon Hydrogen Bond | LEU959 | | 2.10984 | Hydrogen Bond | Hydrogen Bond |
| LEU828 | 2.74989 | Hydrogen Bond | Carbon Hydrogen Bond | LEU959 | | 2.56689 | Hydrogen Bond | Carbon Hydrogen Bond |
| GLY829 | 2.86019 | Hydrogen Bond | Carbon Hydrogen Bond | PHE958 | | 2.82504 | Hydrogen Bond | Carbon Hydrogen Bond |
| CYS909 | 3.01662 | Hydrogen Bond | Hydrogen Bond | ARG1007 | | 2.85652 | Hydrogen Bond | Carbon Hydrogen Bond |
| ASP967 | 3.74146 | Electrostatic | Pi-Anion | LEU881 | | 2.91499 | Hydrophobic | Pi-Sigma |
| LEU956 | 4.16801 | Hydrophobic | Pi-Alkyl | ALA906 | | 3.66596 | Hydrophobic | Pi-Alkyl |
| ALA853 | 4.33593 | Hydrophobic | Pi-Alkyl | LEU1010 | | 4.31732 | Hydrophobic | Pi-Alkyl |
| CYS909 | 4.53848 | Hydrophobic | Alkyl | LEU1010 | | 4.35689 | Hydrophobic | Pi-Alkyl |
| ALA966 | 4.64916 | Hydrophobic | Pi-Alkyl | ALA906 | | 4.41943 | Hydrophobic | Pi-Alkyl |
| VAL836 | 4.83282 | Hydrophobic | Pi-Alkyl | VAL889 | | 4.78439 | Hydrophobic | Pi-Alkyl |
| ALA966 | 4.85219 | Hydrophobic | Amide-Pi Stacked | MET956 | | 5.274 | Other | Pi-Sulfur |
| VAL836 | 4.87551 | Hydrophobic | Pi-Alkyl | VAL938 | | 5.33877 | Hydrophobic | Pi-Alkyl |
| VAL836 | 5.07415 | Hydrophobic | Pi-Alkyl | VAL889 | | 5.42062 | Hydrophobic | Pi-Alkyl |
| MET902 | 5.55814 | Other | Pi-Sulfur |  | |  |  |  |
| ZINC73069247_JAK3 | Distance | Category | Types | ZINC79189223 _JAK3 | | Distance | Category | Types |
| ASN954 | 2.67266 | Hydrogen Bond | Carbon Hydrogen Bond | LEU905 | | 2.34451 | Hydrogen Bond | Hydrogen Bond |
| ASP949 | 2.75197 | Hydrogen Bond | Carbon Hydrogen Bond | GLU903 | | 2.37349 | Hydrogen Bond | Carbon Hydrogen Bond |
| LEU828 | 2.80891 | Hydrogen Bond | Carbon Hydrogen Bond | ALA966 | | 2.43358 | Hydrogen Bond | Carbon Hydrogen Bond |
| GLY829 | 2.85741 | Hydrophobic | Pi-Sigma | GLY829 | | 2.46447 | Hydrogen Bond | Carbon Hydrogen Bond |
| LEU828 | 2.98126 | Hydrogen Bond | Carbon Hydrogen Bond | ARG953 | | 2.58193 | Hydrogen Bond | Carbon Hydrogen Bond |
| LYS830 | 3.41906 | Hydrophobic | Amide-Pi Stacked | ASN954 | | 2.91746 | Hydrogen Bond | Carbon Hydrogen Bond |
| ASP967 | 3.48167 | Electrostatic | Pi-Anion | LEU905 | | 2.94864 | Hydrogen Bond | Hydrogen Bond |
| ASP967 | 3.5196 | Electrostatic | Pi-Anion | ASP967 | | 3.35725 | Electrostatic | Pi-Anion |
| LYS830 | 3.74516 | Hydrophobic | Amide-Pi Stacked | LYS830 | | 3.6706 | Hydrophobic | Amide-Pi Stacked |
| VAL836 | 5.2764 | Hydrophobic | Pi-Alkyl | LEU828 | | 4.01452 | Hydrophobic | Pi-Alkyl |
| VAL836 | 5.37731 | Hydrophobic | Pi-Alkyl | LEU956 | | 4.08592 | Hydrophobic | Pi-Alkyl |
|  | | | | | ALA853 | 4.28251 | Hydrophobic | Pi-Alkyl |
|  |  |  |  |  | LEU956 | 4.31691 | Hydrophobic | Pi-Alkyl |
|  |  |  |  |  | ALA853 | 4.48515 | Hydrophobic | Pi-Alkyl |
|  |  |  |  |  | ALA966 | 4.59901 | Hydrophobic | Pi-Alkyl |
|  |  |  |  |  | VAL836 | 4.64475 | Hydrophobic | Pi-Alkyl |
|  |  |  |  |  | LEU828 | 4.7066 | Hydrophobic | Pi-Alkyl |
|  |  |  |  |  | VAL836 | 4.78358 | Hydrophobic | Alkyl |
|  |  |  |  |  | LEU956 | 4.845 | Hydrophobic | Pi-Alkyl |
|  |  |  |  |  | GLY829 | 5.43927 | Hydrophobic | Amide-Pi Stacked |
|  |  |  |  |  | ALA966 | 5.4401 | Hydrophobic | Alkyl |
|  |  |  |  |  | MET902 | 5.53678 | Other | Pi-Sulfur |
