# Supplementary material for: Revealing innovative JAK1 and JAK3 inhibitors: a comprehensive study utilizing QSAR, 3D-Pharmacophore screening, molecular docking, molecular dynamics, and MM/GBSA analyses
Source: Front Mol Biosci. 2024 Mar 7;11:1348277. doi: 10.3389/fmolb.2024.1348277 (PMC10956358; doi:10.3389/fmolb.2024.1348277)
Supplement: Supplementary file 1 [file DataSheet1.zip › Supplementry File/Supplementry File.pdf]

Table S1 Inhibitory Activity of Cyanamide-Based JAK1 and JAK3 Compounds: Conversion of IC50 to pIC50 Values.

Compound number	SMILES	pIC_{50}(JAK1)	pIC_{50}(JAK3)
1	c1cc(cc(c1)S(=O)(=O)N[C@@H]2C[C@@H](c3c2ccc(c3)c4c5cc[nH]c5ncn4)NC#N)F	5.57	7.97
2	c1cc(cc(c1)S(=O)(=O)N[C@@H]2C[C@@H](c3c2ccc(c3)c4c5cc[nH]c5ncn4)NC#N)C#N	5.77	7.86
3	COc1ccc(cc1)S(=O)(=O)N[C@@H]2C[C@@H](c3c2ccc(c3)c4c5cc[nH]c5ncn4)NC#N	5.76	7.8
4	COCCc1c[nH]c2c1c(ncn2)c3cccc(c3)NC(=O)C=C	4	7.51
5	C[C@H]1CC[C@H](CN1C(=O)C=C)Nc2c3cc[nH]c3ncn2	4.01	7.48
6	CS(=O)(=O)N[C@@H]1C[C@@H](c2c1ccc(c2)c3c4cc[nH]c4ncn3)NC#N	5.72	7.42
7	Cn1c(ccn1)S(=O)(=O)N[C@@H]2C[C@@H](c3c2ccc(c3)c4c5cc[nH]c5ncn4)NC#N	5.4	7.42
8	c1ccc(cc1)c2c[nH]c3c2c(ncn3)c4ccc5c(c4)[C@H](CC5)NC#N	5.48	7.31
9	COCCc1c[nH]c2c1c(ncn2)c3ccc4c(c3)N(CCO4)C(=O)C=C	4	7.31
10	C[C@@]1(CCc2c1cc(cc2)c3c4cc[nH]c4ncn3)NC#N	7.24	7.25
11	c1cc(cc(c1)c2c[nH]c3c2c(ncn3)c4ccc5c(c4)[C@H](CC5)NC#N)CO	4.02	7.11
12	C[C@]1(CCc2c1cc(cc2)c3c4cc[nH]c4ncn3)NC#N	7.03	7.04
13	COCCS(=O)(=O)N[C@@H]1C[C@@H](c2c1ccc(c2)c3c4cc[nH]c4ncn3)NC#N	5.06	6.95
14	c1cc2c(cc1c3c4cc[nH]c4ncn3)[C@H](C[C@H]2O)NC#N	4.14	6.79
15	c1cc2c(cc1c3c4cc[nH]c4ncn3)[C@H](CC2)NC#N	6.19	6.59
16	c1cc2c(cc1c3c4cc[nH]c4ncn3)[C@H]([C@@H](C2)O)NC#N	5.9	6.55
17	COCCc1c[nH]c2c1c(ncn2)c3ccc4c(c3)N(CC(O4)(F)F)C#N	4	6.34
18	C[C@H]1Cc2ccc(cc2[C@@H]1NC#N)c3c4cc[nH]c4ncn3	6.15	6.22
19	c1cc2c(cc1c3c4cc[nH]c4ncn3)[C@H](CC2)NC#N	6.04	6.15
20	c1cc2c(cc1c3c4cc[nH]c4ncn3)[C@H](CCO2)NC#N	5.92	6.11
21	c1cc2c(cc1c3c4cc[nH]c4ncn3)[C@H](CCO2)NC#N	5.93	5.92
22	COCCc1c[nH]c2c1c(ncn2)c3ccc4c(c3)N(CCO4)C#N	5.19	5.91
23	c1cc2c(cc1c3c4cc[nH]c4ncn3)[C@H](C[C@@H]2O)NC#N	5.96	5.87
24	C[C@@H]1Cc2ccc(cc2[C@@H]1NC#N)c3c4cc[nH]c4ncn3	6.01	5.82
25	COCCc1c[nH]c2c1c(ncn2)c3ccc4c(c3)[C@H](CC4)NC#N	5.12	5.8
26	c1cc2c(cc1c3c4cc[nH]c4ncn3)[C@@H](CC2)NC#N	6.07	5.24
27	COCCc1c[nH]c2c1c(ncn2)c3ccc4c(c3)[C@H](CCO4)NC#N	5.42	5.23
28	c1cc2c(cc1c3c4cc[nH]c4ncn3)[C@@H](CCO2)NC#N	6.18	5.21
29	CN(C#N)[C@H]1CCc2c1cc(cc2)c3c4cc[nH]c4ncn3	5.12	5

Table S2 Pharmacophore Models Performance Summary.

**ADRRR**
Enrichment Report<br>-----------------<br>Actives file: hvalidation_5-actives.txt<br>Results: hvalidation_5-ADRRR_1-hits.csv<br>Total actives: 22<br>Total ligands(actives+decoys): 28<br>Number of ranked actives: 22<br>BEDROC(alpha=160.9, alpha*Ra=126.4214): 1.000

BEDROC(alpha=20.0, alpha*Ra=15.7143): 0.997
BEDROC(alpha=8.0, alpha*Ra=6.2857): 0.958
ROC: 0.83
RIE: 1.27
Area under accumulation curve: 0.57
Ave. Number of outranking decoys: 1
Minimum Tc over all active pairs: n/a
Count and percentage of actives in top N% of decoy results.
% Decoys | 1%| 2%| 5%| 10%| 20%|
# Actives | 0| 0| 0| 0| 7|
% Actives | 0.0| 0.0| 0.0| 0.0| 31.8|

Count and percentage of actives in top N% of results.
% Results | 1%| 2%| 5%| 10%| 20%|
# Actives | 0| 0| 1| 2| 5|
% Actives | 0.0| 0.0| 4.5| 9.1| 22.7|
Enrichment Factors with respect to N% sample size.
% Sample | 1%| 2%| 5%| 10%| 20%|
EF | n/a| 1.3| 1.3| 1.3| 1.3|
EF* | inf| inf| inf| 1.9| 1.9|
EF' | inf| inf| inf| 1.9| 1.9|
DEF | n/a| n/a| n/a| n/a| n/a|
DEF* | n/a| n/a| n/a| n/a| n/a|
DEF' | n/a| n/a| n/a| n/a| n/a|
Eff | -1| -1| -1| -1| 0.228|

Enrichment Factors with respect to N% actives recovered.
% Actives | 40%| 50%| 60%| 70%| 80%| 90%| 100%|
EF | 1.1| 1.2| 1.2| 1.2| 1.1| 1.2| 1.1|
EF* | 2.5| 3| 3.5| 4.1| 2.5| 2.7| 2|
EF' | 2.2| 2.4| 2.6| 2.8| 2.9| 2.9| 2.6|
FOD | 0.04| 0.06| 0.08| 0.09| 0.1| 0.1| 0.2|

ADHRRR

Enrichment Report
Actives file: hvalidation_2-actives.txt
Results: hvalidation_2-ADHRR_3-hits.csv
Total actives: 16
Total ligands(actives+decoys): 24
Number of ranked actives: 16
BEDROC (alpha=160.9, alpha*Ra=107.2667): 1.000
BEDROC (alpha=20.0, alpha*Ra=13.3333): 0.893
BEDROC (alpha=8.0, alpha*Ra=5.3333): 0.826
ROC: 0.75
RIE: 1.34
Area under accumulation curve: 0.58
Ave. Number of outranking decoys: 2
Minimum Tc over all active pairs: n/a

```
Count and percentage of actives in top N% of decoy results.
% Decoys |   1%|   2%|   5%| 10%| 20%|
# Actives |    0|    0|    0|    0|    2|
% Actives | 0.0| 0.0|  0.0|  0.0| 12.5|
Count and percentage of actives in top N% of results.
% Results |   1%|   2%|   5%|  10%|  20%|
# Actives |    0|    0|    1|    2|    3|
% Actives |  0.0|  0.0|  6.2| 12.5| 18.8|

Enrichment Factors with respect to N% sample size.
% Sample |     1%|     2%|     5%|    10%|    20%|
EF       |    n/a|    n/a|    1.5|    1.5|    1.2|
EF*      |    inf|    inf|    inf|      1|      2|
EF'      |    inf|    inf|    inf|      1|      2|
DEF      |    n/a|    n/a|    n/a|    n/a|    n/a|
DEF*     |    n/a|    n/a|    n/a|    n/a|    n/a|
DEF'     |    n/a|    n/a|    n/a|    n/a|    n/a|
Eff      |     -1|     -1|     -1|     -1| -0.231|
Enrichment Factors with respect to N% actives recovered.
% Actives |    40%|    50%|    60%|    70%|    80%|    90%|   100%|
EF        |    1.3|    1.3|    1.3|    1.2|    1.2|    1.1|    1.1|
EF*       |      3|      4|    2.5|    2.8|    2.2|    1.8|    1.6|
EF'       |    1.7|      2|    2.1|    2.1|    2.1|      2|    1.9|
FOD       |   0.08|   0.09|    0.1|    0.1|    0.2|    0.2|    0.2|
```

Table S3 Docking Molecular Analysis

ZINC3843186_JAK1*	Distance	Category	Types	ZINC66252348_JAK1	Distance	Category	Types
GLY1020	2.52986	Hydrogen Bond	Carbon Hydrogen Bond	GLU957	2.0706	Hydrogen Bond	Hydrogen Bond
ASN1008	2.67082	Hydrogen Bond	Carbon Hydrogen Bond	GLU883	2.35814	Hydrogen Bond	Hydrogen Bond
ASP1021	2.93495	Electrostatic	Pi-Anion	GLY882	2.45268	Hydrogen Bond	Carbon Hydrogen Bond
LYS908	3.64756	Electrostatic	Pi-Cation	LEU959	2.59484	Hydrogen Bond	Hydrogen Bond
GLU883	3.96507	Hydrophobic	Amide-Pi Stacked	ARG1007	2.60097	Hydrogen Bond	Carbon Hydrogen Bond
LEU1010	4.33175	Hydrophobic	Pi-Alkyl	LEU881	2.63588	Hydrogen Bond	Carbon Hydrogen Bond
ALA906	4.43548	Hydrophobic	Pi-Alkyl	ARG1007	2.74208	Hydrogen Bond	Carbon Hydrogen Bond
LEU881	4.525	Hydrophobic	Pi-Alkyl	ALA906	3.67755	Hydrophobic	Pi-Alkyl

VAL889	5.1063	Hydrophobic	Pi-Alkyl	VAL889	3.88568	Hydrophobic	Alkyl
HIS885	5.15486	Hydrophobic	Pi-Pi T-shaped	LEU1010	4.28849	Hydrophobic	Pi-Alkyl
LEU1010	5.34988	Hydrophobic	Pi-Alkyl	LEU1010	4.35377	Hydrophobic	Pi-Alkyl
				LEU1010	4.43349	Hydrophobic	Alkyl
				LEU881	4.49756	Hydrophobic	Pi-Alkyl
				ALA906	4.68442	Hydrophobic	Pi-Alkyl
				VAL889	4.76604	Hydrophobic	Pi-Alkyl
				LEU881	4.85903	Hydrophobic	Alkyl
				VAL889	5.14851	Hydrophobic	Pi-Alkyl
				MET956	5.30783	Other	Pi-Sulfur
				VAL938	5.37553	Hydrophobic	Pi-Alkyl
ZINC79189223_JAK3*	Distance	Category	Types	ZINC66252131_JAK1	Distance	Category	Types
ASN954	2.44952	Hydrogen Bond	Carbon Hydrogen Bond	GLU957	1.99993	Hydrogen Bond	Hydrogen Bond
GLU903	2.6817	Hydrogen Bond	Carbon Hydrogen Bond	LEU959	2.10984	Hydrogen Bond	Hydrogen Bond
LEU828	2.74989	Hydrogen Bond	Carbon Hydrogen Bond	LEU959	2.56689	Hydrogen Bond	Carbon Hydrogen Bond
GLY829	2.86019	Hydrogen Bond	Carbon Hydrogen Bond	PHE958	2.82504	Hydrogen Bond	Carbon Hydrogen Bond
CYS909	3.01662	Hydrogen Bond	Hydrogen Bond	ARG1007	2.85652	Hydrogen Bond	Carbon Hydrogen Bond
ASP967	3.74146	Electrostatic	Pi-Anion	LEU881	2.91499	Hydrophobic	Pi-Sigma
LEU956	4.16801	Hydrophobic	Pi-Alkyl	ALA906	3.66596	Hydrophobic	Pi-Alkyl
ALA853	4.33593	Hydrophobic	Pi-Alkyl	LEU1010	4.31732	Hydrophobic	Pi-Alkyl
CYS909	4.53848	Hydrophobic	Alkyl	LEU1010	4.35689	Hydrophobic	Pi-Alkyl
ALA966	4.64916	Hydrophobic	Pi-Alkyl	ALA906	4.41943	Hydrophobic	Pi-Alkyl
VAL836	4.83282	Hydrophobic	Pi-Alkyl	VAL889	4.78439	Hydrophobic	Pi-Alkyl
ALA966	4.85219	Hydrophobic	Amide-Pi Stacked	MET956	5.274	Other	Pi-Sulfur
VAL836	4.87551	Hydrophobic	Pi-Alkyl	VAL938	5.33877	Hydrophobic	Pi-Alkyl
VAL836	5.07415	Hydrophobic	Pi-Alkyl	VAL889	5.42062	Hydrophobic	Pi-Alkyl
MET902	5.55814	Other	Pi-Sulfur				
ZINC73069247_JAK3	Distance	Category	Types	ZINC79189223 _JAK3	Distance	Category	Types
ASN954	2.67266	Hydrogen Bond	Carbon Hydrogen Bond	LEU905	2.34451	Hydrogen Bond	Hydrogen Bond
ASP949	2.75197	Hydrogen Bond	Carbon Hydrogen Bond	GLU903	2.37349	Hydrogen Bond	Carbon Hydrogen Bond
LEU828	2.80891	Hydrogen Bond	Carbon Hydrogen Bond	ALA966	2.43358	Hydrogen Bond	Carbon Hydrogen Bond
GLY829	2.85741	Hydrophobic	Pi-Sigma	GLY829	2.46447	Hydrogen Bond	Carbon Hydrogen Bond
LEU828	2.98126	Hydrogen Bond	Carbon Hydrogen Bond	ARG953	2.58193	Hydrogen Bond	Carbon Hydrogen Bond
LYS830	3.41906	Hydrophobic	Amide-Pi Stacked	ASN954	2.91746	Hydrogen Bond	Carbon Hydrogen Bond
ASP967	3.48167	Electrostatic	Pi-Anion	LEU905	2.94864	Hydrogen Bond	Hydrogen Bond
ASP967	3.5196	Electrostatic	Pi-Anion	ASP967	3.35725	Electrostatic	Pi-Anion
LYS830	3.74516	Hydrophobic	Amide-Pi Stacked	LYS830	3.6706	Hydrophobic	Amide-Pi Stacked
VAL836	5.2764	Hydrophobic	Pi-Alkyl	LEU828	4.01452	Hydrophobic	Pi-Alkyl
VAL836	5.37731	Hydrophobic	Pi-Alkyl	LEU956	4.08592	Hydrophobic	Pi-Alkyl

	ALA853	4.28251	Hydrophobic	Pi-Alkyl
	LEU956	4.31691	Hydrophobic	Pi-Alkyl
	ALA853	4.48515	Hydrophobic	Pi-Alkyl
	ALA966	4.59901	Hydrophobic	Pi-Alkyl
	VAL836	4.64475	Hydrophobic	Pi-Alkyl
	LEU828	4.7066	Hydrophobic	Pi-Alkyl
	VAL836	4.78358	Hydrophobic	Alkyl
	LEU956	4.845	Hydrophobic	Pi-Alkyl
	GLY829	5.43927	Hydrophobic	Amide-Pi Stacked
	ALA966	5.4401	Hydrophobic	Alkyl
	MET902	5.53678	Other	Pi-Sulfur
