# Supplementary material for: Revealing innovative JAK1 and JAK3 inhibitors: a comprehensive study utilizing QSAR, 3D-Pharmacophore screening, molecular docking, molecular dynamics, and MM/GBSA analyses
Source: Front Mol Biosci. 2024 Mar 7;11:1348277. doi: 10.3389/fmolb.2024.1348277 (PMC10956358; doi:10.3389/fmolb.2024.1348277)
Supplement: Supplementary file 1 [file DataSheet1.zip › Supplementry File/Supplementry File_1.docx]

Table S1 Internalt Validation Results

| (Internal validation criteria) | | |  |
| --- | --- | --- | --- |
| JAK1 |  |  |  |
| Q2loo: 0.9173 | R2-Q2loo: 0.0301 | RMSEcv: 0.1684 | MAE cv: 0.1457 |
| PRESS cv: 0.6805 | CCC cv: 0.9576 | |  |
| Q2LMO: 0.8871 | R2Yscr: 0.1732 | Q2Yscr: -0.3648 | RMSE AV Yscr: 0.5311 |
| R2Xrnd: | Q2Xrnd: | R2Yrnd: | Q2Yrnd: |
| JAK3 |  |  |  |
| JAK3 | | |  |
| Q2loo: 0.8269 | R2-Q2loo: 0.0801 | RMSE cv: 0.3477 | MAE cv: 0.2922 |
| PRESS cv: 2.9012 | CCC cv: 0.9119 | |  |
| Q2LMO: 0.8094 | R2Yscr: 0.1719 | Q2Yscr: -0.3752 | RMSE AV Yscr: 0.7588 |
| R2Xrnd: | Q2Xrnd: | R2Yrnd: | Q2Yrnd: |

Table S2 External Validation Results

| JAK1 |  |  |  |  |  |
| --- | --- | --- | --- | --- | --- |
| RMSE ext: 0.3887 | | MAE ext: 0.2638 | | PRESS ext: 0.7554 | R2ext: 0.4959 |
| Q2-F1: 0.4702 | | Q2-F2: 0.4641 | | Q2-F3: 0.5593 | CCC ext: 0.6862 |
| r2m aver.: 0.3403 | | r2m delta: 0.1943 | |  |  |
| Calc. external data regr. angle from diagonal: -14.6396° | | | | | |
| Predictions by LOO: | |  |  |  |  |
| Exp(x) vs. Pred(y): R2: 0.9177 | R'2o: 0.9131 | k': 1.0001 | Clos': 0.0050 | r'2m: 0.8556 | |
| Pred(x) vs. Exp(y): R2: 0.9177 | R2o: 0.9174 | k: 0.9990 | Clos: 0.0003 | r2m: 0.9023 | |
| External predictions by model equation: | | | |  |  |
| Exp(x) vs. Pred(y): R2: 0.4959 | R'2o: 0.2361 | k': 1.0079 | Clos': 0.5238 | r'2m: 0.2431 | |
| Pred(x) vs. Exp(y): R2: 0.4959 | R2o: 0.4820 | k: 0.9878 | Clos: 0.0280 | r2m: 0.437 | |
|  | | |  |  |  |
| JAK3 |  |  |  |  |  |
| RMSE ext: 0.9801 | | MAE ext: 0.8879 | | PRESS ext: 4.8025 | R2ext: 0.2763 |
| Q2-F1: 0.0125 | | Q2-F2: -0.1728 | | Q2-F3: -0.3756 | CCC ext: -0.1284 |
| r2m aver.: -0.9390 | | r2m delta: 2.0684 | |  |  |
| Calc. external data regr. angle from diagonal: -48.8187° | | | | | |
| Predictions by LOO: | |  |  |  |  |
| Exp(x) vs. Pred(y): R2: 0.8322 | R'2o: 0.8205 | k': 1.0010 | Clos': 0.0141 | r'2m: 0.7421 | |
| Pred(x) vs. Exp(y): R2: 0.8322 | R2o: 0.8278 | k: 0.9963 | Clos: 0.0053 | r2m: 0.7769 | |
| External predictions by model equation: | | | |  |  |
| Exp(x) vs. Pred(y): R2: 0.2763 | R'2o: -66.0254 | k': 0.9567 | Clos': 239.9983 | r'2m: -1.9732 | |
| Pred(x) vs. Exp(y): R2: 0.2763 | R2o: -0.1532 | k: 1.0207 | Clos: 1.5546 | r2m: 0.0952 | |

Table S3 Scores and Metrics for Various Features Model2 (JAK1 Model).

| Model | Survival Score | Site Score | Vector Score | Volume Score | Selectivity Score | Num Matched | Inactive Score | Adjusted Score | BEDROC Score |
| --- | --- | --- | --- | --- | --- | --- | --- | --- | --- |
| ADRRR_1 | 5.68 | 0.85 | 0.95 | 0.80 | 1.74 | 22 | 2.12 | 3.56 | 1 |
| AADRR_1 | 5.57 | 0.99 | 1 | 0.85 | 1.38 | 22 | 2.70 | 2.87 | 1 |
| AADRR_2 | 5.50 | 0.83 | 0.95 | 0.80 | 1.58 | 22 | 2.16 | 3.34 | 1 |
| DRRR_1 | 5.50 | 0.99 | 1.00 | 0.85 | 1.31 | 22 | 2.69 | 2.81 | 1 |
| AAARR_1 | 5.40 | 0.84 | 0.95 | 0.80 | 1.47 | 22 | 2.12 | 3.29 | 1 |
| ADRR_1 | 5.37 | 0.99 | 1 | 0.85 | 1.19 | 22 | 2.69 | 2.69 | 1 |
| ADRR_2 | 5.34 | 0.99 | 1 | 0.85 | 1.15 | 22 | 2.69 | 2.65 | 1 |
| AARR_1 | 5.30 | 0.99 | 1 | 0.85 | 1.12 | 22 | 2.69 | 2.62 | 1 |
| AAADR_1 | 5.27 | 0.83 | 0.95 | 0.80 | 1.34 | 22 | 2.12 | 3.16 | 1 |
| ADRR_3 | 5.26 | 0.89 | 0.93 | 0.78 | 1.31 | 22 | 2.17 | 3.09 | 1 |
| ADRR_4 | 5.23 | 0.84 | 0.94 | 0.80 | 1.31 | 22 | 2.16 | 3.08 | 1 |
| AAADR_2 | 5.23 | 0.84 | 0.95 | 0.78 | 1.31 | 22 | 2.20 | 3.04 | 1 |
| ARRR_1 | 5.17 | 0.85 | 0.94 | 0.80 | 1.24 | 22 | 2.08 | 3.10 | 1 |
| AADR_1 | 5.17 | 0.99 | 1.00 | 0.85 | 0.98 | 22 | 2.69 | 2.48 | 1 |
| ADRR_5 | 5.19 | 0.85 | 0.94 | 0.79 | 1.27 | 22 | 2.17 | 3.03 | 1 |
| AADR_2 | 5.13 | 1 | 1 | 0.85 | 0.94 | 22 | 2.78 | 2.35 | 1 |

Table S4 Scores and Metrics for Various Features (JAK3 Model).

| Model | Survival Score | Site Score | Vector Score | Volume Score | Selectivity Score | Num Matched | Inactive Score | Adjusted Score | BEDROC Score |
| --- | --- | --- | --- | --- | --- | --- | --- | --- | --- |
| AAADRR_2 | 5.67 | 0.87 | 0.98 | 0.79 | 1.75 | 19 | 2.62 | 3.05 | 0.96 |
| ADRRR_1 | 5.67 | 0.88 | 0.99 | 0.81 | 1.72 | 19 | 2.71 | 2.95 | 0.96 |
| ADRRR_2 | 5.57 | 0.88 | 0.97 | 0.79 | 1.65 | 19 | 2.63 | 2.95 | 0.96 |
| AAADRR_3 | 5.50 | 0.78 | 0.95 | 0.69 | 1.82 | 18 | 2.23 | 3.27 | 0.96 |
| AAADRR_4 | 5.34 | 0.63 | 0.97 | 0.68 | 1.79 | 19 | 2.40 | 2.94 | 0.96 |
| ADHRR_1 | 5.73 | 0.91 | 0.98 | 0.76 | 1.86 | 17 | 2.54 | 3.20 | 0.87 |
| AADDRR_1 | 5.70 | 0.94 | 0.98 | 0.67 | 1.90 | 16 | 2.43 | 3.27 | 0.87 |
| DDRRR_1 | 5.58 | 0.91 | 0.98 | 0.66 | 1.82 | 17 | 2.42 | 3.17 | 0.87 |
| AADDRR_2 | 5.58 | 0.82 | 0.97 | 0.72 | 1.87 | 16 | 2.36 | 3.22 | 0.87 |
| AADDRR_3 | 5.57 | 0.83 | 0.95 | 0.71 | 1.88 | 16 | 2.50 | 3.07 | 0.87 |
| AADHR_1 | 5.55 | 0.90 | 0.98 | 0.76 | 1.68 | 17 | 2.53 | 3.02 | 0.87 |
| AADHR_2 | 5.55 | 0.97 | 1.00 | 0.80 | 1.55 | 17 | 2.74 | 2.81 | 0.87 |
| AADDRR_4 | 5.53 | 0.77 | 0.99 | 0.73 | 1.84 | 16 | 2.48 | 3.05 | 0.87 |
| AAHRR_1 | 5.64 | 0.99 | 1.00 | 0.69 | 1.76 | 16 | 2.54 | 3.10 | 0.83 |

Table S5 ADMET Analysis.

| **Model** | **Result** | **Probability** | **Result** | **Probability** | **Result** | **Probability** | **Result** | **Probability** |
| --- | --- | --- | --- | --- | --- | --- | --- | --- |
| **Absorption** | | | | | | | | |
| **Blood-Brain Barrier** | BBB+ | 0.8412 | BBB+ | 0.9324 | BBB+ | 0.8855 | BBB+ | 0.9067 |
| **Human Intestinal Absorption** | HIA+ | 0.9973 | HIA+ | 1 | HIA+ | 0.995 | HIA+ | 0.9886 |
| **Caco-2 Permeability** | Caco2- | 0.5519 | Caco2- | 0.562 | Caco2- | 0.5376 | Caco2- | 0.6378 |
| **P-glycoprotein Substrate** | Non-substrate | 0.6445 | Non-substrate | 0.6648 | Substrate | 0.7367 | Substrate | 0.6255 |
| **P-glycoprotein Inhibitor** | Non-inhibitor | 0.7975 | Non-inhibitor | 0.7341 | Inhibitor | 0.7588 | Non-inhibitor | 0.5291 |
|  | Non-inhibitor | 0.6467 | Non-inhibitor | 0.7472 | Inhibitor | 0.9202 | Non-inhibitor | 0.6863 |
| **Renal Organic Cation Transporter** | Non-inhibitor | 0.7307 | Non-inhibitor | 0.884 | Inhibitor | 0.5234 | Non-inhibitor | 0.6606 |
| Distribution | | | | | | | | |
| Subcellular localization | Mitochondria | 0.7739 | Mitochondria | 0.7173 | Lysosome | 0.4326 | Plasma membrane | 0.4255 |
| Metabolism | | | | | | | | |
| CYP450 2C9 Substrate | Non-substrate | 0.7007 | Non-substrate | 0.8423 | Non-substrate | 0.8053 | Non-substrate | 0.7576 |
| CYP450 2D6 Substrate | Non-substrate | 0.8106 | Non-substrate | 0.826 | Non-substrate | 0.6949 | Non-substrate | 0.7457 |
| CYP450 3A4 Substrate | Non-substrate | 0.5835 | Substrate | 0.5161 | Substrate | 0.8105 | Substrate | 0.6803 |
| CYP450 1A2 Inhibitor | Inhibitor | 0.8298 | Inhibitor | 0.8746 | Non-inhibitor | 0.8758 | Non-inhibitor | 0.7976 |
| CYP450 2C9 Inhibitor | Inhibitor | 0.6917 | Inhibitor | 0.7297 | Non-inhibitor | 0.845 | Non-inhibitor | 0.7141 |
| CYP450 2D6 Inhibitor | Non-inhibitor | 0.728 | Non-inhibitor | 0.9141 | Non-inhibitor | 0.9287 | Non-inhibitor | 0.8682 |
| CYP450 2C19 Inhibitor | Inhibitor | 0.7456 | Inhibitor | 0.5885 | Non-inhibitor | 0.8199 | Non-inhibitor | 0.761 |
| CYP450 3A4 Inhibitor | Inhibitor | 0.5643 | Non-inhibitor | 0.6297 | Non-inhibitor | 0.855 | Non-inhibitor | 0.8627 |
| CYP Inhibitory Promiscuity | High CYP Inhibitory Promiscuity | 0.9084 | High CYP Inhibitory Promiscuity | 0.7366 | Low CYP Inhibitory Promiscuity | 0.8595 | Low CYP Inhibitory Promiscuity | 0.7378 |
| Excretion | | | | | | | | |
| Toxicity | | | | | | | | |
| Human Ether-a-go-go-Related Gene Inhibition | Weak inhibitor | 0.84 | Weak inhibitor | 0.9942 | Weak inhibitor | 0.8996 | Weak inhibitor | 0.754 |
|  | Non-inhibitor | 0.5229 | Non-inhibitor | 0.9042 | Inhibitor | 0.7489 | Inhibitor | 0.631 |
| AMES Toxicity | Non AMES toxic | 0.5182 | Non AMES toxic | 0.5229 | Non AMES toxic | 0.5471 | Non AMES toxic | 0.5521 |
| Carcinogens | Non-carcinogens | 0.8287 | Non-carcinogens | 0.9113 | Non-carcinogens | 0.8943 | Non-carcinogens | 0.687 |
| Fish Toxicity | High FHMT | 0.9913 | High FHMT | 0.9458 | High FHMT | 0.8579 | High FHMT | 0.769 |
| Tetrahymena Pyriformis Toxicity | High TPT | 0.95 | High TPT | 0.9453 | High TPT | 0.7512 | High TPT | 0.9327 |
| Honey Bee Toxicity | Low HBT | 0.6615 | Low HBT | 0.6163 | Low HBT | 0.8598 | Low HBT | 0.8061 |
| Biodegradation | Not ready biodegradable | 1 | Not ready biodegradable | 0.7176 | Not ready biodegradable | 0.9945 | Not ready biodegradable | 0.9654 |
| Acute Oral Toxicity | III | 0.5771 | III | 0.7048 | III | 0.6707 | III | 0.6339 |
| Carcinogenicity (Three-class) | Non-required | 0.5808 | Non-required | 0.5623 | Non-required | 0.6627 | Non-required | 0.6254 |
| ADMET Predicted Profile --- Regression | | | | | | | | |
| Model | Value | Unit | Value | Unit | Value | Unit | Value | Unit |
| Absorption |  |  |  |  |  |  |  |  |
| Aqueous solubility | -3.4622 | LogS | -3.9019 | LogS | -3.0301 | LogS | -3.554 | LogS |
| Caco-2 Permeability | 1.1066 | LogPapp, cm/s | 0.9019 | LogPapp, cm/s | 0.8707 | LogPapp, cm/s | 0.6248 | LogPapp, cm/s |
| Distribution | | | | | | | | |
| Metabolism | | | | | | | | |
| Excretion | | | | | | | | |
| Toxicity | | | | | | | | |
| Rat Acute Toxicity | 2.4654 | LD50, mol/kg | 2.3404 | LD50, mol/kg | 2.8524 | LD50, mol/kg | 2.6868 | LD50, mol/kg |
| Fish Toxicity | 1.3603 | pLC50, mg/L | 0.5673 | pLC50, mg/L | 1.2943 | pLC50, mg/L | 1.4924 | pLC50, mg/L |
| Tetrahymena Pyriformis Toxicity | 0.626 | pIGC50, ug/L | 0.4018 | pIGC50, ug/L | 0.5709 | pIGC50, ug/L | 0.5255 | pIGC50, ug/L |

Asas

| Model | Result | Probability | Result | Probability | Result | Probability | Result | Probability |
| --- | --- | --- | --- | --- | --- | --- | --- | --- |
| Absorption | | | | | | | | |
| Blood-Brain Barrier | BBB+ | 0.8942 | BBB+ | 0.977 | BBB+ | 0.953 | BBB+ | 0.8006 |
| Human Intestinal Absorption | HIA+ | 1 | HIA+ | 1 | HIA+ | 1 | HIA+ | 1 |
| Caco-2 Permeability | Caco2- | 0.6284 | Caco2- | 0.5129 | Caco2- | 0.5923 | Caco2- | 0.5341 |
| P-glycoprotein Substrate | Substrate | 0.5114 | Substrate | 0.5787 | Substrate | 0.6229 | Non-substrate | 0.8331 |
| P-glycoprotein Inhibitor | Non-inhibitor | 0.7349 | Non-inhibitor | 0.5383 | Non-inhibitor | 0.7396 | Non-inhibitor | 0.7126 |
|  | Non-inhibitor | 0.659 | Inhibitor | 0.5983 | Inhibitor | 0.7344 | Inhibitor | 0.7173 |
| Renal Organic Cation Transporter | Non-inhibitor | 0.7214 | Inhibitor | 0.637 | Non-inhibitor | 0.6338 | Non-inhibitor | 0.8464 |
| Distribution | |  |  |  |  |  |  |  |
| Subcellular localization | Lysosome | 0.3578 | Mitochondria | 0.6712 | Nucleus | 0.4945 | Lysosome | 0.4821 |
| Metabolism | |  |  |  |  |  |  |  |
| CYP450 2C9 Substrate | Non-substrate | 0.8145 | Non-substrate | 0.8298 | Non-substrate | 0.7185 | Non-substrate | 0.729 |
| CYP450 2D6 Substrate | Non-substrate | 0.7719 | Substrate | 0.6126 | Non-substrate | 0.7556 | Non-substrate | 0.7817 |
| CYP450 3A4 Substrate | Substrate | 0.52 | Substrate | 0.5738 | Non-substrate | 0.5052 | Non-substrate | 0.5903 |
| CYP450 1A2 Inhibitor | Non-inhibitor | 0.7872 | Non-inhibitor | 0.6966 | Inhibitor | 0.6706 | Inhibitor | 0.7915 |
| CYP450 2C9 Inhibitor | Non-inhibitor | 0.6075 | Inhibitor | 0.5054 | Non-inhibitor | 0.6675 | Non-inhibitor | 0.5773 |
| CYP450 2D6 Inhibitor | Non-inhibitor | 0.8813 | Non-inhibitor | 0.781 | Non-inhibitor | 0.8758 | Non-inhibitor | 0.8513 |
| CYP450 2C19 Inhibitor | Non-inhibitor | 0.6484 | Inhibitor | 0.8172 | Non-inhibitor | 0.5665 | Inhibitor | 0.5213 |
| CYP450 3A4 Inhibitor | Non-inhibitor | 0.9464 | Non-inhibitor | 0.6943 | Inhibitor | 0.7266 | Non-inhibitor | 0.5498 |
| CYP Inhibitory Promiscuity | Low CYP Inhibitory Promiscuity | 0.8227 | High CYP Inhibitory Promiscuity | 0.8699 | High CYP Inhibitory Promiscuity | 0.6388 | High CYP Inhibitory Promiscuity | 0.7716 |
| Excretion | | | | | | | | |
| Toxicity | | | | | | | | |
| Human Ether-a-go-go-Related Gene Inhibition | Strong inhibitor | 0.5141 | Weak inhibitor | 0.8716 | Weak inhibitor | 0.9877 | Weak inhibitor | 0.9647 |
|  | Non-inhibitor | 0.6296 | Inhibitor | 0.6895 | Non-inhibitor | 0.5363 | Non-inhibitor | 0.7398 |
| AMES Toxicity | Non AMES toxic | 0.5687 | Non AMES toxic | 0.5944 | Non AMES toxic | 0.6169 | Non AMES toxic | 0.716 |
| Carcinogens | Non-carcinogens | 0.7261 | Non-carcinogens | 0.9391 | Non-carcinogens | 0.9549 | Non-carcinogens | 0.8303 |
| Fish Toxicity | High FHMT | 0.7666 | High FHMT | 0.5341 | High FHMT | 0.6987 | High FHMT | 0.5764 |
| Tetrahymena Pyriformis Toxicity | High TPT | 0.7892 | High TPT | 0.7593 | High TPT | 0.849 | High TPT | 0.8604 |
| Honey Bee Toxicity | Low HBT | 0.6908 | Low HBT | 0.8833 | Low HBT | 0.8222 | Low HBT | 0.793 |
| Biodegradation | Not ready biodegradable | 0.9653 | Not ready biodegradable | 0.8932 | Not ready biodegradable | 0.9934 | Not ready biodegradable | 1 |
| Acute Oral Toxicity | III | 0.5774 | III | 0.6414 | III | 0.4593 | III | 0.535 |
| Carcinogenicity (Three-class) | Non-required | 0.5917 | Non-required | 0.6636 | Non-required | 0.6105 | Non-required | 0.6583 |
| ADMET Predicted Profile --- Regression | | | |  |  |  |  |  |
| Model | Value | Unit | Value | Unit | Value | Unit | Value | Unit |
| Absorption |  |  |  |  |  |  |  |  |
| Aqueous solubility | -3.1602 | LogS | -2.6901 | LogS | -3.175 | LogS | -3.4837 | LogS |
| Caco-2 Permeability | 0.3672 | LogPapp, cm/s | 0.6956 | LogPapp, cm/s | 0.6126 | LogPapp, cm/s | 0.5288 | LogPapp, cm/s |
| Distribution | | | | | | | | |
| Metabolism | | | | | | | | |
| Excretion | | | | | | | | |
| Toxicity | | | | | | | | |
| Rat Acute Toxicity | 2.6819 | LD50, mol/kg | 2.6632 | LD50, mol/kg | 2.7612 | LD50, mol/kg | 2.7044 | LD50, mol/kg |
| Fish Toxicity | 1.6818 | pLC50, mg/L | 1.733 | pLC50, mg/L | 1.5701 | pLC50, mg/L | 1.7193 | pLC50, mg/L |
| Tetrahymena Pyriformis Toxicity | 0.3772 | pIGC50, ug/L | 0.4858 | pIGC50, ug/L | 0.6183 | pIGC50, ug/ | 0.6438 | pIGC50, ug/L |

Aax

Table S6 Biological activities of the selected compounds.

| **Tofacitinib**  Pa Pi Activity  0,936 0,001 Janus tyrosine kinase 3 inhibitor  0,909 0,004 Tyrosine kinase inhibitor  0,873 0,001 Janus tyrosine kinase 1 inhibitor  0,857 0,005 Protein kinase inhibitor  0,828 0,002 Janus tyrosine kinase inhibitor  0,732 0,004 Antineoplastic (multiple myeloma)  0,730 0,013 Immunosuppressant  0,541 0,003 Janus tyrosine kinase 2 inhibitor  0,431 0,036 Antipsoriatic  0,390 0,008 Antieczematic atopic  0,403 0,055 Autoimmune disorders treatment  0,321 0,058 Serum-glucocorticoid regulated kinase 1 inhibitor  0,279 0,041 Transplant rejection treatment  0,319 0,089 Analgesic, non-opioid  0,306 0,080 HCV IRES inhibitor  0,297 0,083 H+-transporting two-sector ATPase inhibitor  0,223 0,025 ErbB-1 antagonist  0,242 0,047 Protein-tyrosine kinase p55(blk) inhibitor  0,216 0,032 Antineoplastic (renal cancer)  0,156 0,014 Insulin like growth factor 1 antagonist  0,380 0,241 CYP2H substrate  0,244 0,123 Dermatologic  0,203 0,086 RNA directed DNA polymerase inhibitor  0,176 0,065 Catalase stimulant  0,126 0,016 Insulin growth factor antagonist  0,307 0,201 Neurotransmitter uptake inhibitor  0,147 0,043 Epidermal growth factor receptor kinase inhibitor  0,204 0,101 Polyribonucleotide nucleotidyltransferase inhibitor  0,284 0,187 Glutamate-5-semialdehyde dehydrogenase inhibitor  0,155 0,059 Smooth muscle myosin light chain kinase inhibitor  0,138 0,043 Inosine nucleosidase inhibitor  0,255 0,164 Analgesic  0,137 0,047 Ribosomal protein S6 kinase 1, 70-kDa inhibitor  0,257 0,168 HMGCS2 expression enhancer  0,136 0,050 Growth factor antagonist  0,108 0,022 Protein kinase B inhibitor  0,211 0,132 Pulmonary hypertension treatment  0,095 0,026 Threonine-tRNA ligase inhibitor  0,215 0,150 HIV attachment inhibitor  0,096 0,032 MAP kinase 8 inhibitor  0,100 0,039 Protein kinase B gamma inhibitor  0,203 0,142 Adenosine regulator  0,077 0,018 Rho-associated kinase inhibitor  0,117 0,061 Ribosomal protein S6 kinase inhibitor  0,112 0,059 Dimethylhistidine N-methyltransferase inhibitor  0,114 0,063 Hyperprolactinemia treatment  0,175 0,125 Respiratory distress syndrome treatment  0,112 0,063 Protein-tyrosine kinase Lyn inhibitor  0,107 0,060 ALK inhibitor  0,109 0,062 Constipation treatment  0,111 0,068 Protein-tyrosine kinase (PTK, not ETK, WZC) inhibitor  0,117 0,075 Ribosomal protein S6 kinase, 70-kDa inhibitor  0,105 0,069 Abl kinase inhibitor  0,062 0,027 Phosphatidylinositol 3-kinase delta inhibitor  0,126 0,091 Protein kinase B alpha inhibitor  0,115 0,080 Nicotinamide phosphoribosyltransferase inhibitor  0,059 0,028 Protein kinase B beta inhibitor  0,102 0,072 Ephrin antagonist  0,225 0,196 Inotropic  0,078 0,050 Rho-associated kinase I inhibitor  0,038 0,012 Bradykinin B1 receptor antagonist  0,090 0,064 Protein kinase C inhibitor  0,213 0,189 Raynaud's phenomenon treatment  0,045 0,021 Proto-oncogene tyrosine-protein kinase c-hck inhibitor  0,094 0,075 Allergic rhinitis treatment  0,098 0,082 MAP kinase kinase 7 inhibitor  0,032 0,017 Osteoclast antagonist  0,047 0,032 Epidermal growth factor antagonist  0,108 0,100 Bcr-Abl kinase inhibitor  0,165 0,162 Protein-synthesizing GTPase inhibitor  0,037 0,035 Phosphatidylinositol 3-kinase beta inhibitor  0,087 0,086 Dyrk kinase inhibitor  **A1**  Pa Pi Activity  0,591 0,011 CYP2A11 substrate  0,575 0,014 5 Hydroxytryptamine release inhibitor  0,562 0,052 Mannotetraose 2-alpha-N-acetylglucosaminyltransferase inhibitor  0,557 0,052 Oxidoreductase inhibitor  0,522 0,028 Histamine release inhibitor  0,527 0,048 General pump inhibitor  0,454 0,012 CYP2C9 inhibitor  0,464 0,040 Malate dehydrogenase (acceptor) inhibitor  0,495 0,073 Antineoplastic  0,465 0,061 Lipid metabolism regulator  0,393 0,008 CYP2A10 substrate  0,454 0,074 HIF1A expression inhibitor  0,499 0,137 Antieczematic  0,420 0,071 Antiviral (Rhinovirus)  0,397 0,059 CYP2A4 substrate  0,384 0,059 CYP2F1 substrate  0,375 0,063 Methylenetetrahydrofolate reductase (NADPH) inhibitor  0,390 0,082 4-Nitrophenol 2-monooxygenase inhibitor  0,357 0,054 RELA expression inhibitor  0,355 0,058 Mediator release inhibitor  0,341 0,044 Hydroxylamine reductase (NADH) inhibitor  0,375 0,080 Kinase inhibitor  0,304 0,020 Paraoxonase substrate  0,298 0,032 RNA directed DNA polymerase inhibitor  0,362 0,102 Spasmolytic, urinary  0,354 0,096 Ecdysone 20-monooxygenase inhibitor  0,340 0,089 Vasodilator, coronary  0,328 0,084 Bilirubin oxidase inhibitor  0,342 0,101 Nitrate reductase (cytochrome) inhibitor  0,263 0,049 ATPase stimulant  0,330 0,115 Rubredoxin-NAD+ reductase inhibitor  0,256 0,043 3-Hydroxy-4-oxoquinoline 2,4-dioxygenase inhibitor  0,299 0,088 Antinephritic  0,286 0,078 Vasodilator  0,342 0,136 Antianginal  0,271 0,071 Glycerol-3-phosphate dehydrogenase inhibitor  0,268 0,067 Cholestanetriol 26-monooxygenase inhibitor  0,234 0,037 FMN reductase inhibitor  0,279 0,085 CYP2A5 substrate  0,388 0,195 Antiischemic, cerebral  0,249 0,069 Spasmolytic, Papaverin-like  0,285 0,104 HCV IRES inhibitor  0,386 0,206 CDP-glycerol glycerophosphotransferase inhibitor  0,291 0,112 MAP3K5 inhibitor  0,252 0,074 Clavaminate synthase inhibitor  0,251 0,073 Thyroxine 5-deiodinase inhibitor  0,189 0,013 Histone deacetylase SIRT2 inhibitor  0,194 0,019 Histone deacetylase class III inhibitor  0,241 0,067 Transactivator transcription protein inhibitor  0,315 0,145 2-Dehydropantoate 2-reductase inhibitor  0,258 0,089 Monodehydroascorbate reductase (NADH) inhibitor  0,293 0,127 Centromere associated protein inhibitor  0,199 0,034 Catalase stimulant  0,240 0,079 Cell wall biosynthesis inhibitor  0,249 0,088 2-Hydroxymuconate-semialdehyde hydrolase inhibitor  0,272 0,113 CYP2B5 substrate  0,231 0,073 Thiol protease inhibitor  0,278 0,127 CYP3A4 inducer  0,287 0,149 Fragilysin inhibitor  0,268 0,133 Cyclic AMP phosphodiesterase inhibitor  0,237 0,108 tRNA-pseudouridine synthase I inhibitor  0,321 0,194 Complement factor D inhibitor  0,258 0,134 CYP3A inducer  0,294 0,171 Platelet derived growth factor receptor kinase inhibitor  0,208 0,086 Alcohol dehydrogenase [NAD(P)+] inhibitor  0,204 0,089 Cyclooxygenase substrate  0,201 0,085 CYP1A2 inducer  0,275 0,163 CYP2A1 substrate  0,233 0,123 Spasmolytic  0,269 0,164 3-Hydroxybenzoate 6-monooxygenase inhibitor  0,282 0,179 CYP2C19 inducer  0,171 0,072 GST M1-1 substrate  0,314 0,216 5-O-(4-coumaroyl)-D-quinate 3'-monooxygenase inhibitor  0,107 0,009 Poly(ADP-ribose) polymerase 2 inhibitor  0,257 0,160 N-acetylneuraminate 7-O(or 9-O)-acetyltransferase inhibitor  0,157 0,065 Testosterone 17beta-dehydrogenase inhibitor  0,270 0,183 Antimyopathies  0,168 0,086 Complement inhibitor  0,175 0,095 Antiuremic  0,219 0,142 RNA synthesis inhibitor  0,149 0,071 Alcohol dehydrogenase (NADP+) inhibitor  0,167 0,090 Gout treatment  0,114 0,038 Estrogen receptor beta antagonist  0,297 0,222 27-Hydroxycholesterol 7alpha-monooxygenase inhibitor  0,260 0,185 CYP2C12 substrate  0,259 0,185 Calcium regulator  0,149 0,077 Smooth muscle myosin light chain kinase inhibitor  0,116 0,044 Liver cirrhosis treatment  0,128 0,059 Myocardial infarction treatment  0,221 0,156 Nitrite reductase [NAD(P)H] inhibitor  0,121 0,058 Azobenzene reductase inhibitor  0,162 0,100 Nitrate reductase inhibitor  0,184 0,122 Glutathione dehydrogenase (ascorbate) inhibitor  0,233 0,172 NAD(P)+-arginine ADP-ribosyltransferase inhibitor  0,216 0,155 P-benzoquinone reductase (NADPH) inhibitor  0,113 0,052 Estrogen antagonist  0,226 0,165 H+-transporting two-sector ATPase inhibitor  0,181 0,121 Antineoplastic enhancer  0,125 0,066 Transcription factor NF kappa B inhibitor  0,285 0,228 Thromboxane B2 antagonist  0,106 0,050 Protein kinase C inhibitor  0,095 0,039 Ornithine decarboxylase inhibitor  0,102 0,046 Cytokine release inhibitor  0,159 0,103 Antineoplastic (thyroid cancer)  0,326 0,271 Phosphatase inhibitor  0,273 0,218 Glyceryl-ether monooxygenase inhibitor  0,281 0,227 TP53 expression enhancer  0,136 0,083 UGT2B18 substrate  0,244 0,191 GABA aminotransferase inhibitor  0,273 0,221 Chloride peroxidase inhibitor  0,087 0,037 NAD(P)H dehydrogenase (quinone) inhibitor  0,170 0,124 Severe acute respiratory syndrome treatment  0,056 0,011 Electron transport complex I inhibitor  0,178 0,134 Hemostatic  0,302 0,258 Platelet adhesion inhibitor  0,192 0,151 Antifibrinolytic  0,236 0,196 Radiosensitizer  0,073 0,034 Steroid DELTA-isomerase inhibitor  0,124 0,086 Aryl-alcohol dehydrogenase (NADP+) inhibitor  0,066 0,028 Protein kinase C epsilon inhibitor  0,285 0,247 Antineoplastic (non-Hodgkin's lymphoma)  0,181 0,145 Lipoprotein lipase stimulant  0,208 0,172 Creatininase inhibitor  0,245 0,211 Antipruritic, allergic  0,201 0,169 Isopenicillin-N epimerase inhibitor  0,051 0,021 Sirtuin inhibitor  0,157 0,127 RET inhibitor  0,198 0,169 Cyclomaltodextrinase inhibitor  0,208 0,180 CYP3A7 substrate  0,335 0,308 CYP2H substrate  0,062 0,036 TEC kinase inhibitor  0,144 0,117 Deoxyribonuclease I inhibitor  0,223 0,197 CYP2E1 inducer  0,181 0,156 Glutaminyl-peptide cyclotransferase inhibitor  0,158 0,134 Antineoplastic (sarcoma)  0,174 0,151 Transcription factor inhibitor  0,289 0,267 Testosterone 17beta-dehydrogenase (NADP+) inhibitor  0,044 0,022 Sirtuin 2 inhibitor  0,179 0,157 Hepatoprotectant  0,145 0,124 Vitamin-K-epoxide reductase (warfarin-insensitive) inhibitor  0,160 0,142 Fumarate reductase (NADH) inhibitor  0,106 0,090 Skin whitener  0,195 0,179 Cytochrome-b5 reductase inhibitor  0,054 0,040 Protein kinase C eta inhibitor  0,148 0,134 Mucorpepsin inhibitor  0,078 0,064 Antineoplastic antibiotic  0,133 0,121 CYP2C8 inducer  0,035 0,025 Ryanodine receptor antagonist  0,035 0,025 Ryanodine receptor 1 antagonist  0,064 0,055 Protein kinase C beta inhibitor  0,043 0,036 Microtubule stabilization  0,092 0,085 Succinate dehydrogenase inhibitor  0,079 0,072 Macrophage migration inhibitory factor inhibitor  0,172 0,166 Lysostaphin inhibitor  0,116 0,110 Ca2+/calmodulin-dependent kinase I inhibitor  0,109 0,105 Endoglycosylceramidase inhibitor  0,126 0,124 DNA directed RNA polymerase inhibitor  **A2**  Pa Pi Activity  0,880 0,002 Janus tyrosine kinase 3 inhibitor  0,876 0,004 Tyrosine kinase inhibitor  0,835 0,001 Janus tyrosine kinase 1 inhibitor  0,819 0,005 Protein kinase inhibitor  0,770 0,002 Janus tyrosine kinase inhibitor  0,681 0,004 Antineoplastic (multiple myeloma)  0,688 0,018 Immunosuppressant  0,597 0,013 Autoimmune disorders treatment  0,538 0,003 Janus tyrosine kinase 2 inhibitor  0,537 0,018 Antipsoriatic  0,430 0,013 Transplant rejection treatment  0,410 0,023 Rheumatoid arthritis treatment  0,386 0,008 Antieczematic atopic  0,359 0,064 Dermatologic  0,329 0,052 Serum-glucocorticoid regulated kinase 1 inhibitor  0,295 0,064 Inflammatory Bowel disease treatment  0,305 0,080 HCV IRES inhibitor  0,300 0,082 H+-transporting two-sector ATPase inhibitor  0,234 0,024 ErbB-1 antagonist  0,213 0,005 Rho-associated kinase inhibitor  0,401 0,215 CYP2H substrate  0,163 0,005 Rho-associated kinase II inhibitor  0,139 0,017 Insulin like growth factor 1 antagonist  0,314 0,193 Neurotransmitter uptake inhibitor  0,202 0,087 RNA directed DNA polymerase inhibitor  0,177 0,065 Catalase stimulant  0,220 0,116 Pulmonary hypertension treatment  0,202 0,104 Polyribonucleotide nucleotidyltransferase inhibitor  0,115 0,020 Insulin growth factor antagonist  0,137 0,047 Ribosomal protein S6 kinase 1, 70-kDa inhibitor  0,134 0,045 Inosine nucleosidase inhibitor  0,136 0,050 Epidermal growth factor receptor kinase inhibitor  0,107 0,023 Protein kinase B inhibitor  0,276 0,197 Glutamate-5-semialdehyde dehydrogenase inhibitor  0,183 0,113 Respiratory distress syndrome treatment  0,124 0,055 Growth factor antagonist  0,205 0,138 Adenosine regulator  0,093 0,027 Threonine-tRNA ligase inhibitor  0,215 0,150 HIV attachment inhibitor  0,244 0,179 Transcription factor stimulant  0,244 0,179 Transcription factor NF kappa B stimulant  0,248 0,183 HMGCS2 expression enhancer  0,121 0,060 Lck kinase inhibitor  0,098 0,042 Protein kinase B gamma inhibitor  0,115 0,062 Ribosomal protein S6 kinase inhibitor  0,088 0,041 MAP kinase 8 inhibitor  0,112 0,068 Hyperprolactinemia treatment  0,229 0,187 Inotropic  0,115 0,077 Ribosomal protein S6 kinase, 70-kDa inhibitor  0,184 0,149 Platelet aggregation inhibitor  0,104 0,069 Ephrin antagonist  0,103 0,069 Dimethylhistidine N-methyltransferase inhibitor  0,102 0,071 ALK inhibitor  0,058 0,031 Protein kinase B beta inhibitor  0,113 0,086 Nicotinamide phosphoribosyltransferase inhibitor  0,123 0,098 Protein kinase B alpha inhibitor  0,056 0,034 Phosphatidylinositol 3-kinase delta inhibitor  0,103 0,082 Constipation treatment  0,098 0,078 Protein-tyrosine kinase Lyn inhibitor  0,074 0,055 Rho-associated kinase I inhibitor  0,092 0,076 Dyrk kinase inhibitor  0,096 0,082 Protein-tyrosine kinase (PTK, not ETK, WZC) inhibitor  0,032 0,019 Osteoclast antagonist  0,039 0,027 Proto-oncogene tyrosine-protein kinase c-hck inhibitor  0,097 0,085 MAP kinase kinase 7 inhibitor  0,045 0,034 Epidermal growth factor antagonist  0,095 0,085 Src kinase inhibitor  0,167 0,157 Protein-synthesizing GTPase inhibitor  0,163 0,155 Antiinfertility, female  0,089 0,083 Abl kinase inhibitor  0,073 0,067 Gastric emptying disorders treatment  0,219 0,213 Antineoplastic  0,068 0,062 Cyclin-dependent kinase 9 inhibitor  0,089 0,084 Allergic rhinitis treatment |
| --- |
